# Hupehenine suppresses tumor cell phenotypes and modulates macrophage marker profiles in a diabeticmimetic colorectal cancer model

**DOI:** 10.3389/fphar.2026.1962620

**Published:** 2026-10-20

**Authors:** Ming Qi Zhu, Zi Tong Shi, Hong Mei Hu, Qi Dong Xue, Xin Yu Gu, Xin Cheng Li, Qiu Yan Wang, Yan Zhao, You Wei Zhang, Ji Zhong Zhao

**Affiliations:** 1 Xuzhou Clinical College of Xuzhou Medical University, Xuzhou Central Hospital, Xuzhou, China; 2 Lab of Cell Biology, School of Life Sciences, Jiangsu Normal University, Xuzhou, Jiangsu, China

**Keywords:** colorectal cancer, CXCR2, diabetes, hupehenine, macrophage polarization, polypharmacology, tumor microenvironment

## Abstract

**Background:**

Diabetes is associated with increased colorectal cancer (CRC) risk and mortality, but the molecular links remain incompletely understood. We aimed to characterize shared molecular features of diabetes and CRC, and identify compounds capable of modulating the candidate axis at this interface.

**Methods:**

Shared disease-associated genes were identified using integrated bioinformatics and protein-protein interaction network analysis, followed by analyses of TCGA and single-cell RNA-sequencing data. Deep learning-based (DeepPurpose) and structure-based (AutoDock Vina) virtual screening were used to prioritize multi-target ligands. Binding was assessed by surface plasmon resonance (SPR) and molecular dynamics simulations. Cellular effects were evaluated in a diabetic-mimetic model using high glucose, insulin, and palmitic acid (three factors; TF), with HCT116 cells and THP-1-derived macrophages.

**Results:**

The IL1β-CXCL8-CXCR2 axis was prioritized as a candidate inflammatory module at the diabetes-CRC interface. TCGA and single-cell RNA-seq analyses associated axis-related expression with immune infiltration and macrophage polarization-related transcriptional signatures; higher axis expression was also associated with longer overall survival. Hupehenine (HU), a steroidal alkaloid, was identified as a multi-target ligand engaging IL1β, CXCL8, and CXCR2. SPR showed concentration-dependent binding (KD: CXCR2, 505 nM; CXCL8, 2.2 μM; IL1β, 12.7 μM) and interference with CXCL8-CXCR2 binding. In a diabetic-mimetic model, HU reduced HCT116 cell viability, migration, and invasion. TF-conditioned cells were more sensitive to HU than non-TF cells, whereas a corresponding increase in sensitivity was not observed for 5-fluorouracil or oxaliplatin. HU also decreased M2-associated and increased M1-associated macrophage markers.

**Conclusion:**

These findings support the IL1β-CXCL8-CXCR2 axis as a candidate immunomodulatory module at the diabetes-CRC interface and identify HU as an *in vitro* multi-target ligand. The observed changes in macrophage markers and differential sensitivity under diabetic-mimetic conditions warrant further mechanistic and preclinical evaluation, including direct assessment of macrophage function.

## 1 Introduction

Type 2 diabetes is associated with increased colorectal cancer (CRC) risk, an association supported by epidemiological studies and Mendelian randomization evidence (Ke et al., 2024; Lega and Lipscombe, 2020). With cancer representing a major cause of death among people with diabetes (Collier et al., 2020; The Lancet Diabetes Endocrinology, 2025), understanding the molecular pathways through which the diabetic milieu, including hyperglycemia, hyperinsulinemia, and chronic low-grade inflammation, promotes colorectal carcinogenesis is an important priority. Current therapies do not selectively address this interface: antidiabetic agents such as metformin have shown inconsistent chemopreventive benefits (Shahrivar et al., 2025; Sirtori et al., 2024), and a diabetes-specific benefit of conventional chemotherapeutics, including 5-fluorouracil and oxaliplatin, has not been established. Therapeutic strategies targeting the molecular intersection of diabetic metabolic stress and CRC therefore warrant investigation.

Chronic inflammation has emerged as a unifying mechanism underlying the pathogenesis of both diabetes and cancer (Lega and Lipscombe, 2020; Furman et al., 2019). The diabetic state promotes a systemic pro-inflammatory milieu through hyperglycemia-driven activation of the NLRP3 inflammasome, accumulation of advanced glycation end products (AGEs), and dysregulated adipokine secretion (Wan et al., 2022). This persistent inflammatory environment fuels tumor initiation and progression by enhancing cellular proliferation, survival, angiogenesis, and metastatic dissemination, while simultaneously impairing antitumor immune surveillance (Nishida and Andoh, 2025). Within this pathological crosstalk, two inflammatory mediators are particularly relevant. Interleukin-1β (IL1β) is a pro-inflammatory cytokine that can promote tumor cell proliferation and invasion, and serves as a key upstream inducer of secondary inflammatory cascades (Rébé and Ghiringhelli, 2025). C-X-C motif chemokine ligand 8 (CXCL8; also known as IL-8), acting through its receptor CXCR2, can promote epithelial-mesenchymal transition (EMT), recruits immunosuppressive myeloid cells including neutrophils and myeloid-derived suppressor cells (MDSCs), and maintains cancer stem cell properties (Huang et al., 2023; Li et al., 2021). Importantly, IL1β is a well-established transcriptional inducer of CXCL8 via NF-κB and MAPK/AP-1 pathways (Mizuno et al., 2022), linking upstream inflammatory signaling to downstream chemokine activity. The IL1β-CXCL8-CXCR2 axis therefore represents a rational therapeutic target at the nexus of metabolic inflammation and tumor immunity, yet its specific role in diabetes-associated CRC remains incompletely characterized.

The concept of polypharmacology, simultaneously engaging multiple nodes within a pathogenic network, has attracted interest as a strategy that may improve efficacy and limit compensatory resistance (Diwanji et al., 2023). In inflammatory signaling, inhibitors of IL-1 signaling (e.g., anakinra) or CXCR2 have been investigated in cancer treatment settings (Isambert et al., 2018; Rahimizadeh et al., 2025), yet monotherapy may be limited by redundancy within the cytokine network. Natural products, with their inherent structural complexity and capacity for polypharmacological interactions, offer an attractive source for multi-target drug discovery. Hupehenine (HU) is a steroidal alkaloid found predominantly in the bulbs of *Fritillaria hupehensis* (Hubei Fritillary), a plant traditionally used in Chinese medicine (Duan et al., 2023; Zhang et al., 2008). Recent investigations have revealed biological activities of HU: anti-leishmanial action through inhibition of dephospho-coenzyme A kinase (Menpadi et al., 2023), and neuroprotective potential via inhibition of α-synuclein aggregation (Ghanem et al., 2021). However, the potential of HU as a multi-target modulator at the diabetes-CRC interface remains poorly characterized.

Here, we prioritize the IL1β-CXCL8-CXCR2 axis as a candidate inflammatory signaling module at the diabetes-CRC interface. We examine its associations with immune infiltration and macrophage polarization-related signatures in CRC datasets. We further show that hupehenine, a steroidal alkaloid from *F. hupehensis*, binds all three proteins *in vitro*, reduces tumor cell viability and motility, and modulates macrophage markers in a diabetic-mimetic model. These findings provide a rationale for further investigating this axis as a potential link between metabolic stress and the CRC immune microenvironment.

## 2 Materials and methods

Detailed methods are provided in the Supplementary Material. Brief descriptions of key procedures are given below.

### 2.1 Data acquisition and bioinformatics analysis

Disease-associated genes were retrieved from DisGeNET, TTD, GeneCards, and OMIM using standardized keyword queries. Intersection analysis identified 531 genes co-associated with diabetes and CRC. GO and KEGG enrichment analyses were performed using clusterProfiler (v4.3.2) in R with an adjusted P < 0.05 cutoff. PPI networks were constructed in STRING (v11.5, confidence ≥0.4) and analyzed in Cytoscape (v3.6.0) using MCODE and CytoHubba plugins. TCGA-COAD and READ expression data were analyzed for differential expression and survival associations. For survival analyses, patients were independently stratified into high- and low-expression groups based on the median expression level of each gene; slight variations in sample sizes across different gene analyses reflect this independent stratification combined with minor missing expression values in the dataset. Immune cell infiltration was estimated by deconvolution analysis. An exploratory composite 14-genes (TCF7L2, PPARG, ADIPOQ, LEP, IGF1, IGF2, IRS1, IRS2, SLC30A8, CDKAL1, GCK, PDK4, IL6, TNF) expression signature was used to define diabetes-related expression subgroups; these subgroups were not equivalent to clinically confirmed diabetes status.

### 2.2 Single-cell RNA-seq analysis

Published scRNA-seq data from a CRC atlas (GSE178341; 371,223 cells from 62 patients) were processed using Seurat v4.3.0. After quality control (200–5,000 genes, <20% mitochondrial reads, removal of doublets), data were normalized with SCTransform and clustered using the first 30 principal components (resolution = 0.8). Cell identities were annotated based on canonical marker gene expression and visualized in a bubble plot. Macrophage polarization-associated transcriptional scores (M1: IL1A, IL1Β, TNF, NOS2, CXCL9, CXCL10; M2: IL10, TGFB1, ARG1, MRC1, CD163, CCL22) and CD8^+^ T-cell exhaustion-associated transcriptional scores (PDCD1, CTLA4, LAG3, HAVCR2, TIGIT, TOX) were calculated using the AddModuleScore function in Seurat (v4). Correlation between CXCL8-expressing myeloid cell proportion and polarization scores was assessed by Pearson correlation. Differences between MMRd and MMRp tumors were evaluated using the Mann-Whitney U test.

### 2.3 Virtual screening and molecular simulations

Deep learning-based screening used the DeepPurpose toolkit with Morgan fingerprint encoding. Structure-based screening used AutoDock Vina. Consensus hits were further evaluated by molecular docking with AutoDock4. Molecular dynamics simulations (100 ns) were performed with GROMACS 2024.3 using the CHARMM36-jul2022 force field for the protein and CGenFF for HU. Complexes were solvated in cubic SPC/E water boxes with a 1.0 nm solute-edge distance and neutralized with counter-ions (gmx genion -neutral). After steepest-descent minimization, systems were equilibrated for 100 ps in NVT (300 K, V-rescale) and 100 ps in NPT (1 bar, C-rescale) with position restraints on the protein and ligand, followed by 100 ns of unrestrained production under the same NPT conditions with coordinates saved every 10 ps. LINCS constraints, PME electrostatics and a 1.2 nm Verlet cutoff (force-switch 1.0–1.2 nm for van der Waals) were used throughout. RMSD, RMSF, Rg, ligand-protein minimum distances (minimum-image and unwrapped), PCA-based free-energy landscapes and MM/GBSA binding free energies (gmx_MMPBSA 1.6.4) were calculated as described in the Supplementary Methods.

### 2.4 Surface plasmon resonance

SPR measurements were performed on a Biacore 1K instrument with proteins immobilized on CM5 sensor chips. Kinetic parameters were derived by fitting to a 1:1 Langmuir binding model. Interference with protein binding to CXCR2 was assessed by pre-saturating CXCR2 surfaces with 10 μM HU.

### 2.5 Cell culture and diabetic-mimetic model

HCT116 and SW480 cells were procured from the American Type Culture Collection (ATCC, Manassas, VA, United States); HCEC-1CT cells were acquired from YaJi Biological (Shanghai, China). HCT116, SW480, and HCEC-1CT cells were cultured in DMEM with 10% FBS. The diabetic-mimetic “three factors” (TF) condition consisted of high glucose (25 mM), insulin (100 nM), and palmitic acid (200 μM). THP-1 monocytes were differentiated into macrophages with PMA (50 ng/mL, 48 h). Macrophage marker profiles were assessed by flow cytometry for CD86 and CD206 and by qRT-PCR for polarization markers.

### 2.6 Functional assays

Cell viability was assessed using the MTT assay after 48 h of drug treatment. IC_50_ values were determined by nonlinear regression analysis of dose-response curves. For chronic TF exposure experiments, cells were cultured under TF conditions for 1, 2, or 3 weeks before functional assays. Migration and invasion were assessed by wound healing and Transwell assays. Transcript and protein abundance were assessed by qRT-PCR and western blotting, respectively. ELISA was used to quantify secreted IL1β and CXCL8. Gene overexpression and siRNA-mediated knockdown were performed using standard protocols. Co-immunoprecipitation used purified recombinant proteins.

### 2.7 Comparative pharmacological assays

HU was compared with the CXCR2 antagonist navarixin (30 μM; MedChemExpress, HY-10198) and the IL1β receptor antagonist anakinra (30 μM; GlpBio, GC39339), alone or in combination, in HCT116 cells under TF conditions. Cell viability was measured by MTT assay at 48 h.

### 2.8 Statistical analysis

Unless otherwise specified, experimental data are presented as mean ± SD from three independent replicates. Normality was assessed using the Shapiro-Wilk test. For data meeting the normality criterion, comparisons used Student’s t-test (two groups) or one-way ANOVA with Tukey’s *post hoc* test (multiple groups). Correlations were assessed by Pearson correlation as indicated, and differences in cell composition or score distributions between groups were assessed by the Mann-Whitney U test. A P value <0.05 was considered statistically significant, with significance levels denoted as follows: ns (not significant), *P < 0.05, **P < 0.01, and ***P < 0.001.

## 3 Results

### 3.1 An inflammatory gene module is shared between diabetes and CRC

To systematically identify the molecular determinants shared between diabetes and CRC, we performed an integrated bioinformatic screen across four disease-gene databases: DisGeNET, TTD, GeneCards, and OMIM. This analysis yielded 1,043 diabetes-associated genes and 3,632 CRC-associated genes (Supplementary Table S1), with an intersection of 531 genes concurrently implicated in both diseases (Figures 1A–C; Supplementary Table S2). Gene Ontology (GO) enrichment analysis revealed that these shared genes were predominantly involved in the regulation of inflammatory responses (Biological Processes), were enriched at the external side of the plasma membrane (Cellular Components), and displayed mRNA base-pairing translational repressor activity among other molecular functions (Figures 1D–F; Supplementary Table S8). Kyoto Encyclopedia of Genes and Genomes (KEGG) pathway analysis further identified the PI3K/AKT signaling pathway, microRNAs in cancer, and the AGE-RAGE signaling pathway in diabetic complications as the most significantly enriched routes (Figure 1G; Supplementary Table S9). An integrative network integrating the shared genes with their enriched functional terms provided a systems-level view of the molecular landscape at the diabetes-CRC interface (Figure 1H).

**FIGURE 1:**
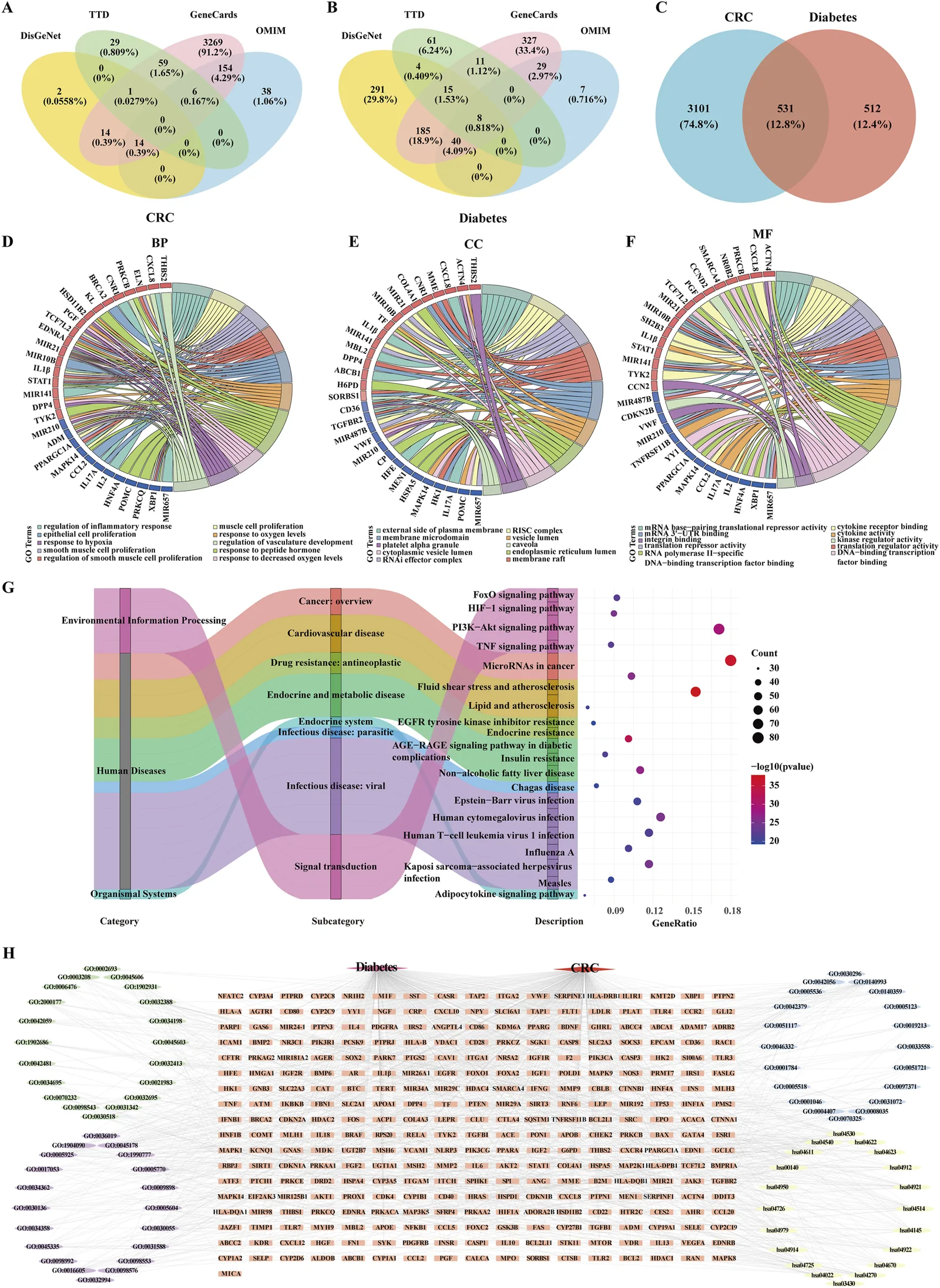
Landscape of genes shared between diabetes and colorectal cancer. **(A,B)** Venn diagrams quantifying CRC-associated **(A)** and diabetes-associated **(B)** gene targets curated from four databases: DisGeNET, TTD, GeneCards, and OMIM. **(C)** Intersection identifying 531 genes co-associated with both diseases. **(D–F)** Gene Ontology enrichment analysis of shared genes: Biological Processes **(D)**, Cellular Components **(E)**, and Molecular Functions **(F)**. Top 30 terms are shown. **(G)** KEGG pathway enrichment analysis. **(H)** Integrated interaction network connecting shared genes with the top 20 enriched terms from each GO category and KEGG pathway.

To pinpoint the candidate network hubs within this shared gene set, we constructed a protein-protein interaction (PPI) network and applied a multi-algorithmic hub-detection strategy. The PPI network, built from the 531 shared genes using STRING and visualized in Cytoscape, revealed dense interconnectivity (Figure 2A). MCODE-based module decomposition identified tightly connected functional clusters and nominated 16 intramodular hub candidates (Figure 2B). To identify consistently highly ranked nodes, we applied four centrality algorithms—Degree, Edge Percolation Component (EPC), Maximum Clique Centrality (MCC), and Maximum Neighborhood Component (MNC)—via CytoHubba (Figures 2C–F). Intersection analysis of the top-ranked genes from all four methods, visualized through an UpSet plot, revealed algorithmic consensus for eight genes: IL1Β, TNF, NFKB1, TLR4, IL6, CXCL8, IFNG, and IL10 (Figure 2G). This core module is dominated by key inflammatory mediators, supporting a potential role for chronic inflammation as a molecular link between diabetes and CRC.

**FIGURE 2:**
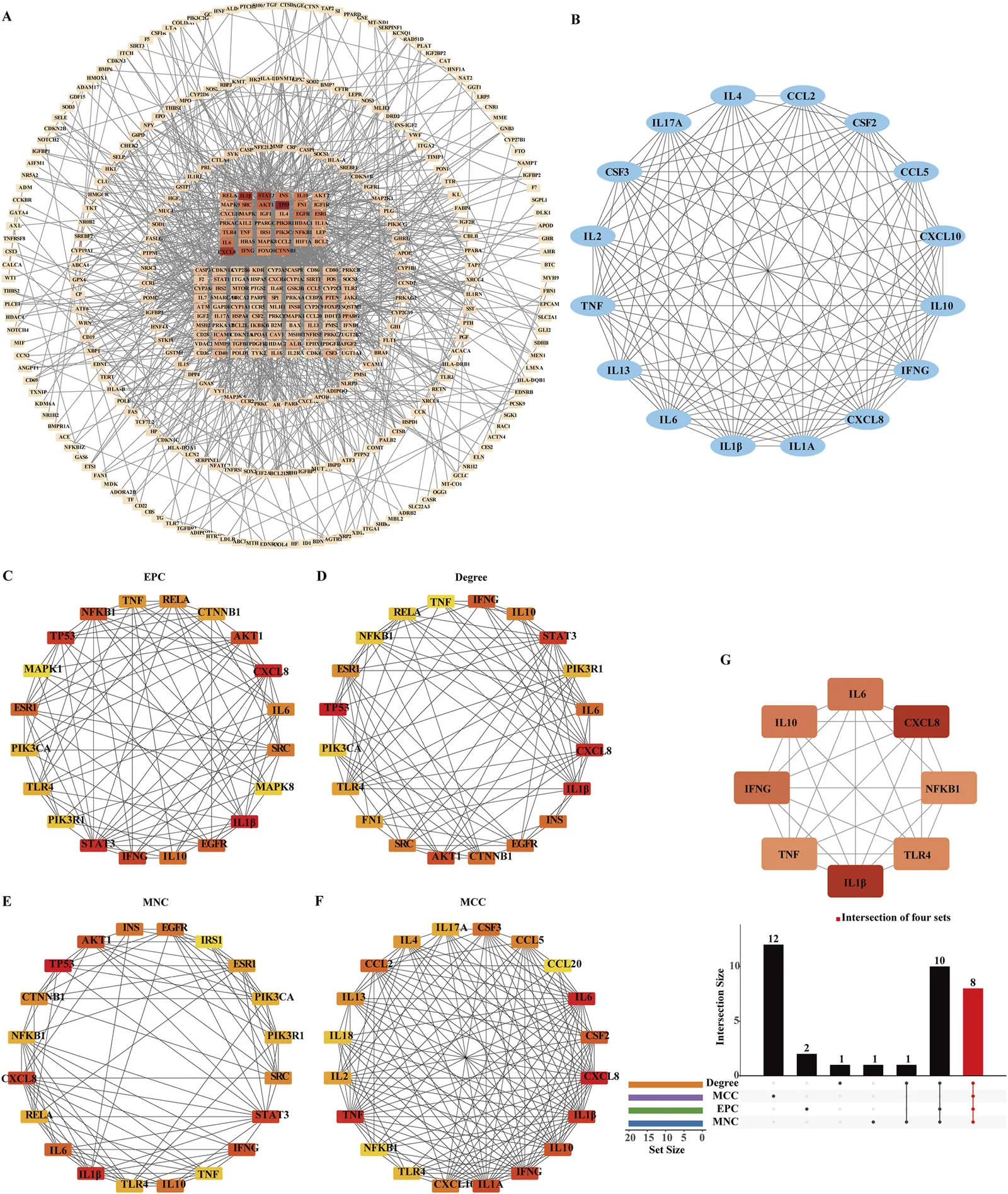
Multi-algorithmic identification of hub genes at the diabetes-CRC interface. **(A)** Protein-protein interaction network of the 531 shared genes. **(B)** MCODE-identified densely connected functional modules. **(C–F)** Top 20 hub genes ranked by Degree **(C)**, EPC **(D)**, MNC **(E)**, and MCC **(F)** centrality algorithms. **(G)** UpSet plot showing consensus of eight core hub genes identified by all four algorithms.

### 3.2 IL1β-CXCL8-CXCR2 axis expression is associated with immune signatures and longer overall survival

To translate these computational predictions into clinical context, we analyzed the TCGA colorectal cancer dataset. Among the eight hub genes, only IL1Β and CXCL8 were significantly upregulated in CRC tumors versus adjacent normal mucosa, while the other six showed no significant differential expression (Figures 3A,B; Supplementary Figure S1A–F). Kaplan-Meier analysis revealed that high expression of either IL1Β or CXCL8 was associated with longer overall survival, whereas the other six hub genes showed no significant association (Figures 3C,D; Supplementary Figure S1G). Patients with concurrent high expression of both IL1Β and CXCL8 exhibited the longest observed overall survival (Figure 3E). Given that IL1β is an established transcriptional inducer of CXCL8 and CXCL8 signals through its cognate receptor CXCR2, we extended the analysis to this receptor. CXCR2 was similarly upregulated in tumors and positively co-expressed with both IL1Β and CXCL8 (Figure 3F; Supplementary Figure S1M,N). To explore diabetic-related transcriptional patterns, we stratified CRC patients into high- and low-score subgroups based on a composite expression signature of 14 diabetes-related genes. High co-expression of all three genes was associated with prolonged overall survival, particularly in patients stratified to the high diabetes-related expression-score subgroup (Figures 3G,H). Immune deconvolution analysis revealed elevated CD8^+^ T cell infiltration scores and a high M2/M1 macrophage ratio in the high-axis-expression subgroup (Figures 3I,J).

**FIGURE 3:**
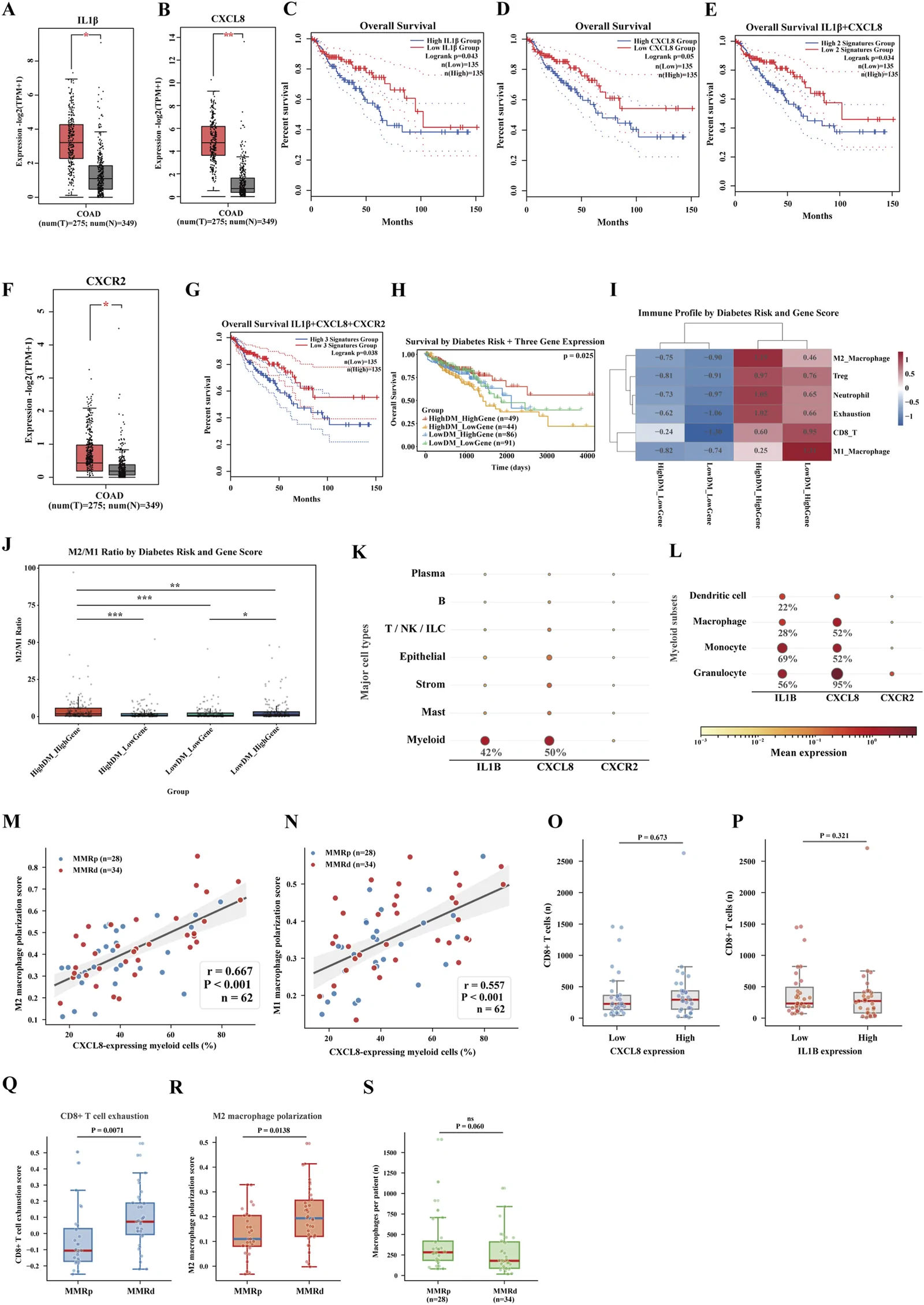
Expression of IL1β-CXCL8-CXCR2 axis genes is associated with immune signatures and longer overall survival. **(A,B)** IL1Β and CXCL8 are significantly upregulated in CRC tumors (n = 275) versus normal mucosa (n = 349; TCGA-COAD and TCGA-READ). **(C–E)** Kaplan-Meier analysis showing prolonged overall survival in patients with high IL1Β **(C)**, CXCL8 **(D)**, or combined **(E)** expression. **(F)** CXCR2 expression in tumors. **(G)** Kaplan-Meier overall survival curves stratified by the three-gene signature (IL1Β, CXCL8, CXCR2). Patients were dichotomized into high- and low-expression groups. Analysis restricted to patients with complete expression data for the three genes (n = 270). **(H)** Kaplan-Meier overall survival curves stratified by combining diabetes-related expression-score status and the three-gene expression signature. Patients were categorized into four groups: HighDM_HighGene, HighDM_LowGene, LowDM_HighGene, and LowDM_LowGene (n = 49, 44, 86, and 91, respectively; total n = 270). **(I)** Immune infiltration heatmap for the high diabetes-related expression-score, high axis-expression subgroup, showing concurrent CD8^+^ T cell and myeloid cell enrichment alongside elevated expression of exhaustion-associated genes. **(J)** Elevated estimated M2/M1 macrophage ratio based on immune deconvolution; this estimate does not directly measure immunosuppressive function. **(K–L)** IL1Β, CXCL8, and CXCR2 mean expression across seven major cell types **(K)** and four myeloid subsets **(L)**. Cell color denotes mean expression level (log scale); numbers indicate the percentage of cells expressing each gene. **(M)** Correlation between the proportion of CXCL8-expressing myeloid cells and M2-associated macrophage transcriptional score. Blue, MMRp tumors (n = 28); red, MMRd tumors (n = 34); r = 0.667, P < 0.001 (Pearson correlation). **(N)** Correlation between the proportion of CXCL8-expressing myeloid cells and M1-associated macrophage transcriptional score. r = 0.557, P < 0.001. **(O–P)** CD8^+^ T cell counts per patient stratified by median CXCL8 **(O)** and median IL1Β **(P)** expression levels. **(Q)** CD8^+^ T-cell exhaustion-associated transcriptional scores in MMRd versus MMRp tumors (P = 0.0071). **(R)** M2-associated macrophage transcriptional scores in MMRd versus MMRp tumors (P = 0.0138). **(S)** Macrophage counts per patient in MMRd versus MMRp tumors (P = 0.06, not significant). *P < 0.05, **P < 0.01, ***P < 0.001.

To explore this apparent paradox and complement the TCGA deconvolution analysis, we analyzed a published single-cell RNA-seq atlas of CRC comprising 371,223 tumor-derived cells from 62 patients (Pelka et al., 2021). Cell cluster identities were confirmed by canonical marker gene expression (Supplementary Figure S1O). IL1Β and CXCL8 were expressed almost exclusively by myeloid cells, including monocytes, granulocytes, and macrophages, while CXCR2 was predominantly detected on granulocytes (Figures 3K,L; Supplementary Figure S2A–D; Supplementary Table S10), suggesting that expression of these genes is enriched in myeloid populations. Across the 62 patients, the proportion of CXCL8-expressing myeloid cells was positively correlated with the M2-associated macrophage transcriptional score (r = 0.667, P < 0.001; Figure 3M), and to a lesser extent with the M1-associated score (r = 0.557, P < 0.001; Figure 3N), indicating associations with both polarization-related signatures; the difference between these correlations was not formally tested. In contrast, CD8^+^ T cell proportions did not differ significantly between patients stratified by IL1Β/CXCL8 expression levels (Figures 3O,P), although this lack of a statistically significant difference does not exclude an association with T-cell recruitment. Stratification by MMR status revealed that MMRd tumors exhibited significantly higher CD8^+^ T-cell exhaustion-associated transcriptional scores (P = 0.0071) and elevated M2-associated macrophage transcriptional scores (P = 0.0138) compared with MMRp tumors (Figures 3Q,R), whereas no statistically significant difference in macrophage counts was detected between MMR groups (P = 0.06, ns; Figure 3S). To further investigate the intercellular communication mediated by this axis, we performed ligand-receptor interaction analysis using CellChat (v1.6.0). The inferred communication patterns involving axis-related genes suggested myeloid-to-myeloid and myeloid-to-granulocyte interactions (Supplementary Figure S2E). These single-cell data associate expression of axis-related genes with macrophage polarization-related signatures but do not directly establish signaling activity, functional polarization, or T-cell suppression. One possible explanation for the longer survival associated with higher axis expression is that it accompanies favorable immune features, such as MMR deficiency and CD8^+^ T-cell infiltration, while also co-occurring with immunoregulatory myeloid programs. The mechanism underlying the survival association remains unresolved. The observed pattern is compatible with an immune-infiltrated microenvironment that also expresses inhibitory immune markers (Chen and Mellman, 2017), providing a hypothesis for subsequent functional testing.

### 3.3 Virtual screening identifies hupehenine as a multi-target ligand for the IL1β-CXCL8-CXCR2 axis

Having prioritized the IL1β-CXCL8-CXCR2 axis as a candidate signaling module, we sought to identify natural compounds capable of simultaneously engaging all three targets. We employed a tiered computational screening strategy combining deep learning and structure-based methods (Figure 4A). The MCE natural product library was screened using DeepPurpose, a deep-learning framework for drug-target interaction prediction, which ranked compounds by their predicted multi-target affinity scores against IL1β, CXCL8, and CXCR2. In parallel, structure-based virtual screening was performed using AutoDock Vina. Cross-referencing the top 30 candidates from each method yielded a consensus set of four compounds, among which Hupehenine (HU, HY-N0413) had the highest predicted affinity scores across all three targets (Figures 4B–D).

**FIGURE 4:**
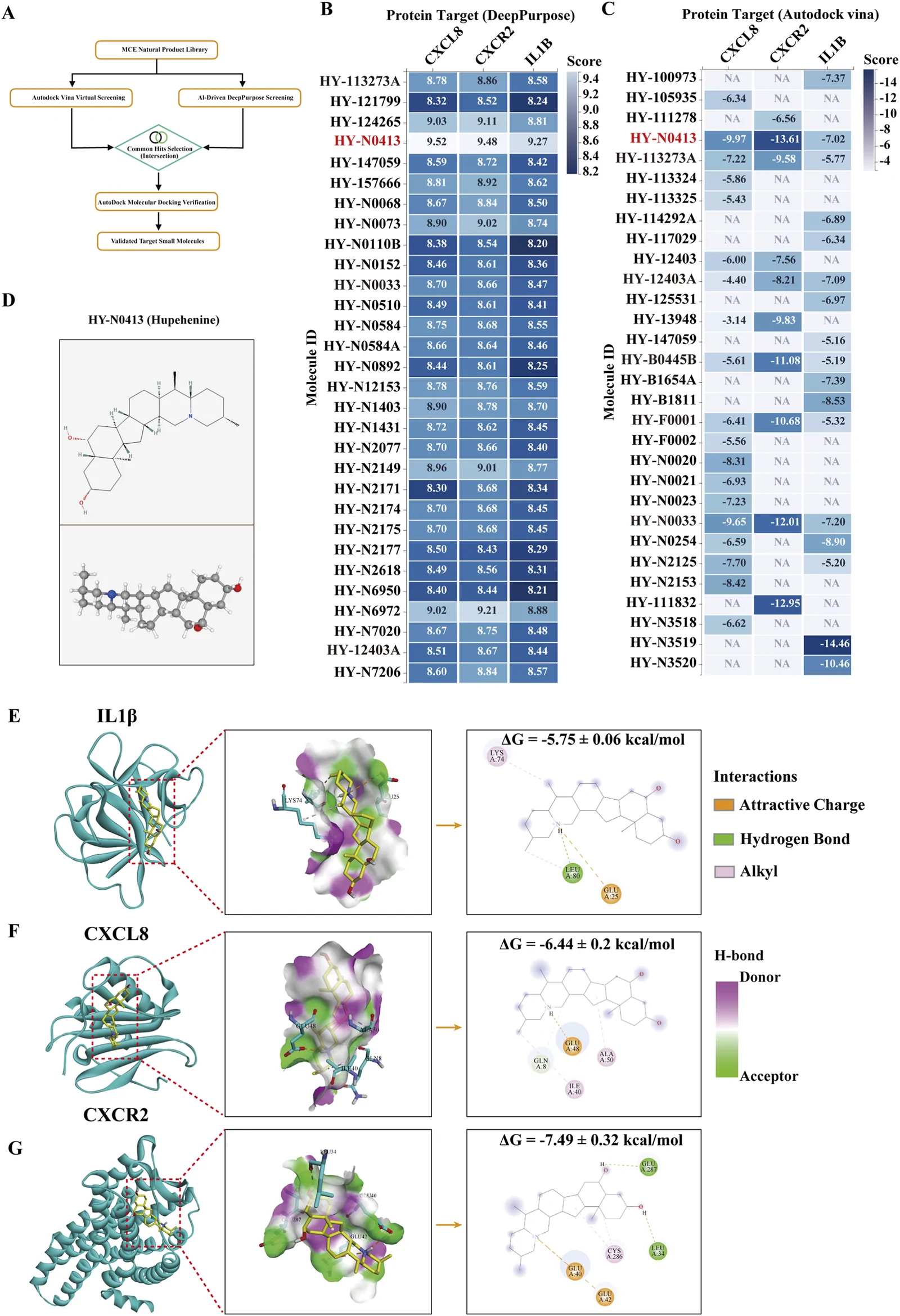
Virtual screening identifies Hupehenine as a multi-target ligand. **(A)** Schematic of the integrated screening strategy combining DeepPurpose (deep learning) and AutoDock Vina (structure-based) methods. **(B,C)** Consensus screening heatmaps highlighting HU as the top-ranked multi-target candidate. **(D)** Chemical structure of HU. **(E–G)** Molecular docking poses and interaction diagrams of HU bound to IL1β **(E)**, CXCL8 **(F)**, and CXCR2 **(G)**, with calculated binding affinities and key residue contacts.

Molecular docking simulations with AutoDock4 predicted favorable docking scores for HU with each target protein. Three independent docking runs yielded docking scores of −5.75 ± 0.06 kcal/mol for IL1β, −6.44 ± 0.20 kcal/mol for CXCL8, and −7.49 ± 0.32 kcal/mol for CXCR2 (Figures 4E–G; Supplementary Table S11). Analysis of the predominant binding poses indicated putative contacts involving a network of conventional hydrogen bonds, carbon-hydrogen bonds, and extensive hydrophobic contacts, including π-alkyl, π-anion, and π-cation interactions, with key residues (e.g., LYS, ALA, GLU, LEU) within the respective binding pockets (Figures 4E–G). The more favorable docking score for CXCR2 suggested a potential binding preference that required experimental evaluation.

### 3.4 Biophysical analyses support HU binding to multiple targets and interference with CXCL8-CXCR2 binding

To further evaluate the predicted complexes, we first performed 100-ns all-atom molecular dynamics simulations (MDS) for each HU-protein complex. Protein backbones showed limited deviation from the reference structure in all three systems (protein-backbone RMSD <0.39 nm; Figures 5A–C), whereas ligand RMSD in the protein-aligned frame is reported separately and may reflect changes in ligand conformation, orientation, position, or binding status (Figures 5A–C). Using both minimum-image and unwrapped distances to distinguish apparent dissociation from periodic-image artifacts (Figures 5D–F), HU remained continuously bound to CXCR2 for 100% of frames but detached from CXCL8 after ∼91 ns and repeatedly dissociated from IL1β from ∼53 ns onward. Ligand retention in these individual trajectories followed the order CXCR2 ≫ CXCL8 > IL1β, qualitatively consistent with the docking-score ranking. RMSF showed low fluctuations in the folded cores, with larger fluctuations confined to terminal and loop regions (Figures 5G–I); Rg showed limited variation (Supplementary Figure S3A–C); and PCA-derived free-energy landscapes were more compact for CXCR2 than for CXCL8 or IL1β (Figures 5J–O). MM/GBSA gave ΔG = −18.9 ± 2.6 kcal/mol for CXCR2, −6.3 ± 1.6 kcal/mol for CXCL8, and −3.5 ± 1.9 kcal/mol for IL1β (Supplementary Figure S3D); however, full-trajectory averages for CXCL8 and IL1β are affected by dissociation and cannot be interpreted as bound-state affinity estimates for the initial poses. As each system was simulated as a single 100-ns trajectory, replicate simulations will be required to establish full ensemble convergence.

**FIGURE 5:**
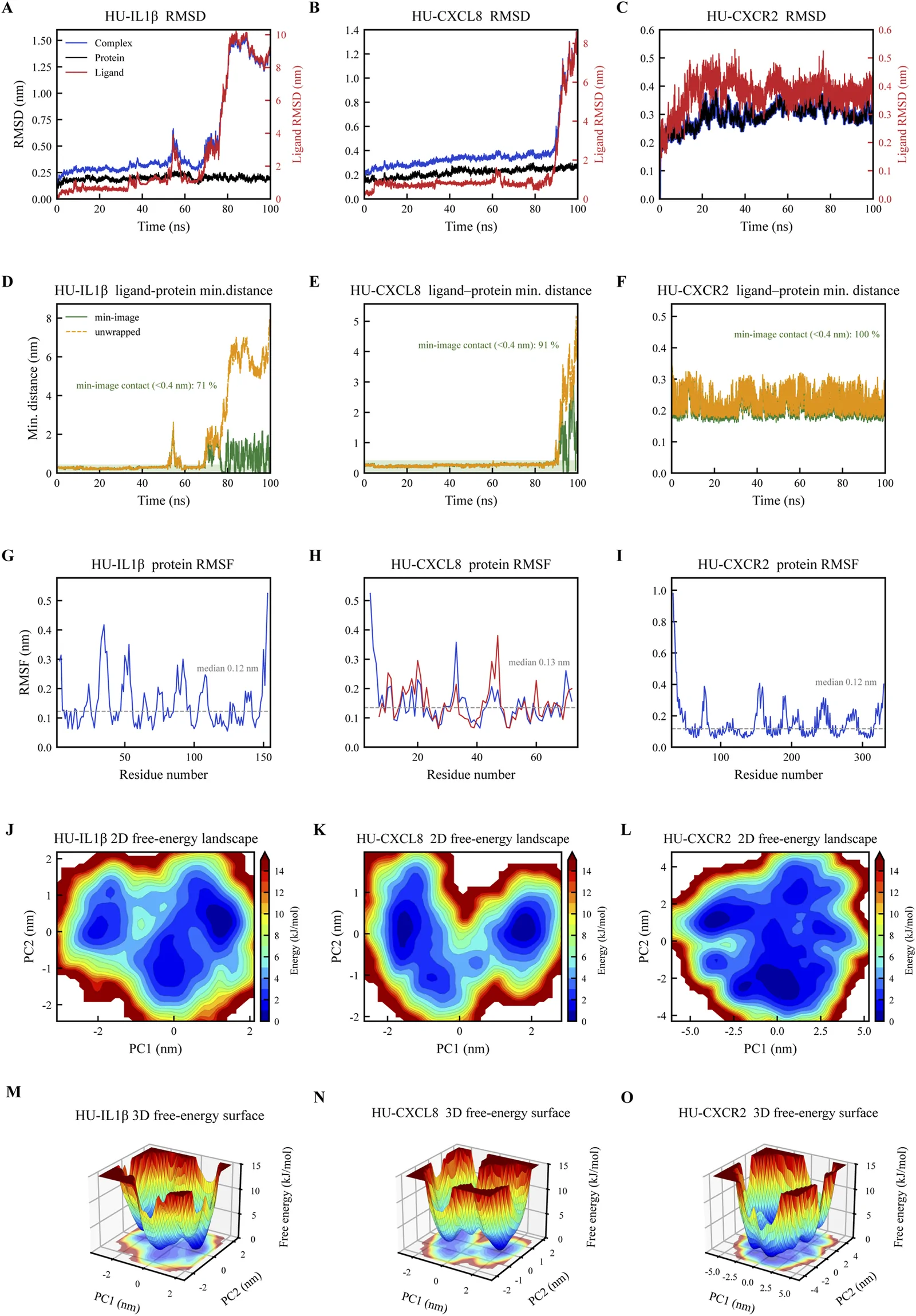
Molecular dynamics simulations of HU complexes with IL1β, CXCL8 and CXCR2. **(A–C)** Backbone (black) and complex (blue) RMSD after superposition on the protein backbone; the right axis (red) shows ligand RMSD in the protein frame. **(D–F)** Minimum ligand-protein distance (solid, minimum-image; dashed, unwrapped). HU remained bound to CXCR2 throughout the simulation, dissociated from CXCL8 after approximately 91 ns, and showed repeated dissociation from IL1β from approximately 53 ns onward. **(G–I)** Per-residue backbone RMSF (median, dashed line); CXCL8 chains A and B in blue and red. **(J–O)** PCA-derived Gibbs free-energy landscapes as 2D contour maps **(J,L,N)** and 3D surfaces **(K,M,O)** for IL1β, CXCL8 and CXCR2. Analyses cover 0–100 ns.

We next employed surface plasmon resonance (SPR) spectroscopy for direct, label-free measurement of HU binding. HU bound to all three purified targets in a dose-dependent manner (Figures 6A–C), with KD values of 505.3 ± 4.6 nM for CXCR2, 2.2 ± 0.1 μM for CXCL8, and 12.7 ± 1.0 μM for IL1β, indicating the strongest measured affinity for CXCR2. This concordance should be interpreted in light of the distinct quantities being compared: docking provides a scoring-function estimate, MD describes the behavior of an initial pose within a finite trajectory, and SPR estimates binding affinity from the measured sensorgrams. Nevertheless, the persistent CXCR2 engagement in MD agrees with the strongest SPR affinity, and the micromolar SPR affinities of CXCL8 and IL1β are consistent with their weaker and transient binding in MD. In additional protein-binding assays, IL1β bound CXCR2 with KD = 16.2 ± 0.76 nM and CXCL8 bound CXCR2 with KD = 1.5 ± 0.13 nM (Figures 6D,E). Critically, pre-saturation of the CXCR2-immobilized surface with 10 μM HU inhibited subsequent binding of both IL1β and CXCL8 by over 10-fold, shifting apparent KD values into the micromolar range (Figures 6F,G). These results support interference with binding to the immobilized CXCR2 preparation but do not, by themselves, establish an orthosteric competitive mechanism. Together, these assays support *in vitro* binding of HU to all three tested proteins with differing affinities, with the strongest measured affinity for CXCR2 and evidence of interference with CXCL8-CXCR2 binding.

**FIGURE 6:**
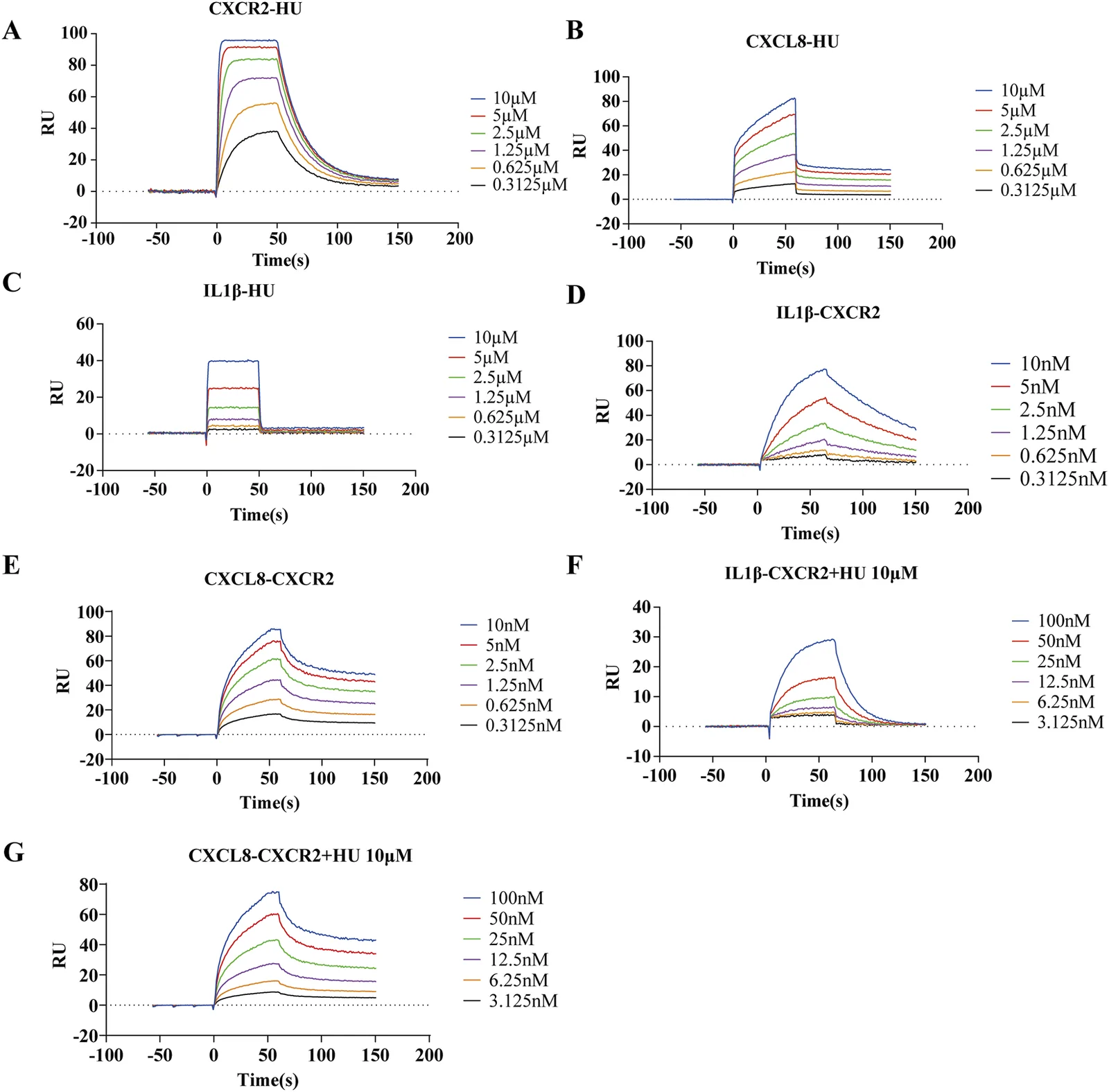
SPR analysis of HU binding to multiple targets and interference with binding to CXCR2. **(A–C)** Dose-dependent binding sensorgrams of HU to CXCR2 (**(A)**; KD = 505.3 nM), CXCL8 (**(B)**; KD = 2.2 μM), and IL1β (**(C)**; KD = 12.7 μM). **(D,E)** Protein-binding assays: IL1β-CXCR2 (**(D)**; KD = 16.2 nM) and CXCL8-CXCR2 (**(E)**; KD = 1.5 nM). **(F,G)** Binding-interference assays: HU pre-saturation of CXCR2 reduces subsequent IL1β **(F)** and CXCL8 **(G)** binding, shifting apparent KD to the micromolar range.

### 3.5 HU counteracts tumor-associated phenotypes under diabetic-mimetic conditions

To model selected metabolic features relevant to diabetes in CRC cells, we treated HCT116 cells with a combination of high glucose, insulin, and palmitic acid (collectively termed “three factors”, TF) to mimic the key metabolic stressors in diabetes. Chronic exposure to TF for 1, 2, and 3 weeks significantly and time-dependently increased the viability, migration, and invasion of HCT116 cells, as assessed by MTT, wound healing, and Transwell assays, respectively (Figures 7A–F). TF also increased viability in SW480 cells (Supplementary Figure S4A). HU reduced these TF-associated readouts, including the viability, migration, and invasion in cells chronically exposed to TF for 3 weeks (Figures 7G–L). Notably, HCT116 cells conditioned in the long-term TF microenvironment exhibited heightened sensitivity to HU, with a lower half-maximal inhibitory concentration (IC_50_: 26.7 ± 1.3 μM, Figure 7M) compared to cells maintained under standard conditions (IC_50_: 72.2 ± 3.6 μM, Figure 7N). SW480 cells also exhibited increased sensitivity to HU under TF conditions (Supplementary Figure S4B). In contrast, TF conditioning was not associated with increased sensitivity to conventional chemotherapeutics: the IC_50_ for 5-fluorouracil was 31.2 ± 2.8 μM (TF) versus 30.2 ± 1.5 μM (non-TF) (Figures 7O–Q), and for oxaliplatin was 5.2 ± 0.4 μM (TF) versus 4.5 ± 0.6 μM (non-TF) (Figures 7R–T). Furthermore, HU did not significantly reduce the viability of non-tumorigenic human colonic epithelial cells (HCEC-1CT) at concentrations up to 200 μM (Figure 7U). Collectively, these results indicate that TF exposure enhances tumor-associated phenotypes in this *in vitro* model and increases cellular sensitivity to HU. The findings in HCEC-1CT cells suggest a potential *in vitro* selectivity window.

**FIGURE 7:**
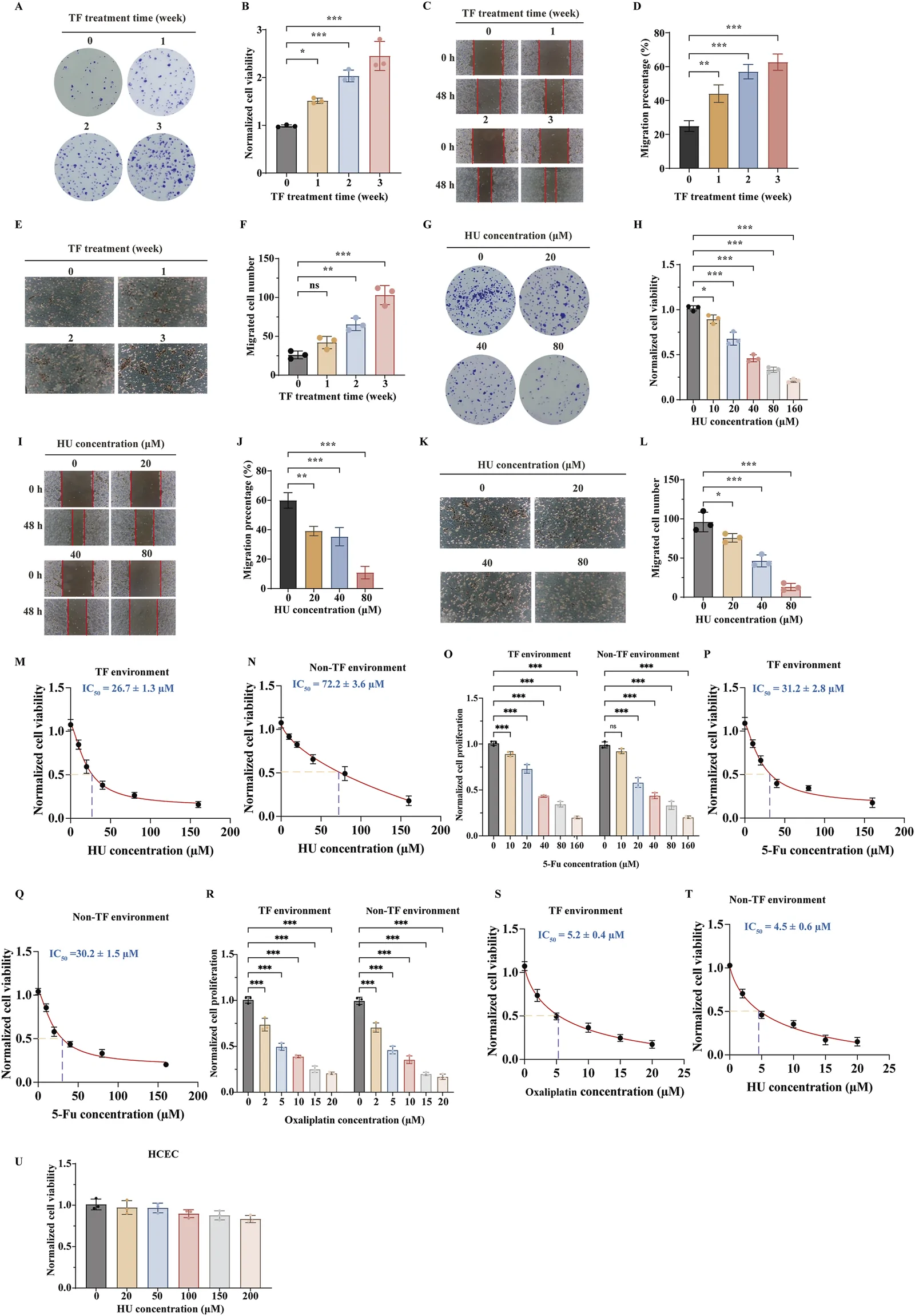
HU reduces TF-associated cellular phenotypes and shows increased activity in TF-conditioned cells. **(A–F)** Chronic TF exposure (1–3 weeks) time-dependently enhances HCT116 cell viability **(A,B)**, migration **(C,D)**, and invasion **(E,F)**. **(G–L)** HU dose-dependently suppresses the TF-enhanced cell viability **(G,H)**, migration **(I,J)**, and invasion **(K,L)**. **(M,N)** TF-conditioned cells show enhanced HU sensitivity (IC_50_ = 26.7 μM vs. 72.2 μM in non-TF; determined by MTT assay at 48 h). **(O–T)** TF does not increase sensitivity to 5-fluorouracil **(O–Q)**; IC_50_: 31.2 ± 2.8 μM TF vs. 30.2 ± 1.5 μM non-TF) or oxaliplatin (R-T; IC_50_: 5.2 ± 0.4 μM TF vs. 4.5 ± 0.6 μM non-TF); all IC_50_ values were determined by MTT assay at 48 h under identical conditions. **(U)** HU does not significantly reduce HCEC-1CT viability at concentrations ≤200 μM under the tested conditions. *P < 0.05, **P < 0.01, ***P < 0.001.

### 3.6 HU modulates axis-associated signaling and interferes with CXCL8–CXCR2 binding

The diabetic-mimetic TF microenvironment increased axis-associated expression readouts, as shown by the marked upregulation of upstream and axis-associated components (NF-κB p65, IL1β, CXCL8, and CXCR2) and downstream effectors (PI3K, AKT, mTOR) (Figures 8A,B; Supplementary Figure S4C), as well as secreted IL1β and CXCL8 (Figure 8C). HU treatment selectively counteracted the TF-induced increase in CXCL8, suppressing mRNA expression and secretion while sparing the total protein pool (Figures 8A–C; Supplementary Figure S4C), indicating that transcript, intracellular protein, and secreted protein levels did not change in parallel. Concurrently, it attenuated the measured downstream PI3K/AKT/mTOR pathway readouts (Figures 8A,B), while leaving NF-κB p65, IL1β, and CXCR2 largely unaffected (Figures 8A,B). siRNA-mediated knockdown of IL1Β produced changes similar to those observed with HU treatment, reducing CXCL8 expression and downstream signaling (Figure 8D; Supplementary Figure S5A), supporting a contribution of IL1β to the measured pathway responses. Nevertheless, HU remained effective even in the presence of exogenous IL1β (Figure 8E), suggesting that HU can inhibit the measured responses despite exogenous IL1β stimulation. Pre-incubation of recombinant IL1β with HU reduced its ability to induce CXCL8 and downstream pathway activation (Figure 8F), consistent with inhibition of IL1β-induced responses, although residual free HU could also act on downstream targets. Knockdown of CXCL8 or CXCR2 (Supplementary Figure S5B,C) similarly suppressed the PI3K/AKT/mTOR cascade (Figure 8G), and HU reduced pathway signaling driven by exogenous CXCL8 or CXCR2 overexpression (Figure 8H; Supplementary Figure S5D,E). The stimulatory activity of CXCL8 was abrogated when the chemokine was pre-incubated with HU, or when cells were pre-treated with HU before CXCL8 exposure (Figure 8I). Co-immunoprecipitation with purified proteins demonstrated that HU disrupted the physical interaction between CXCL8 and CXCR2 (Figure 8J). To further define the level at which HU intercepts axis signaling, we employed gain-of-function approaches. NF-κB p65 overexpression increased axis-associated readouts and HCT116 cell proliferation and migration, effects that were attenuated by HU (Supplementary Figure S5F; Supplementary Figure S6A–D). In contrast, PI3K/AKT overexpression (Supplementary Figure S5G,H) largely bypassed HU-mediated inhibition (Supplementary Figure S6E–G), supporting an effect upstream of PI3K/AKT in this experimental system without excluding other targets.

**FIGURE 8:**
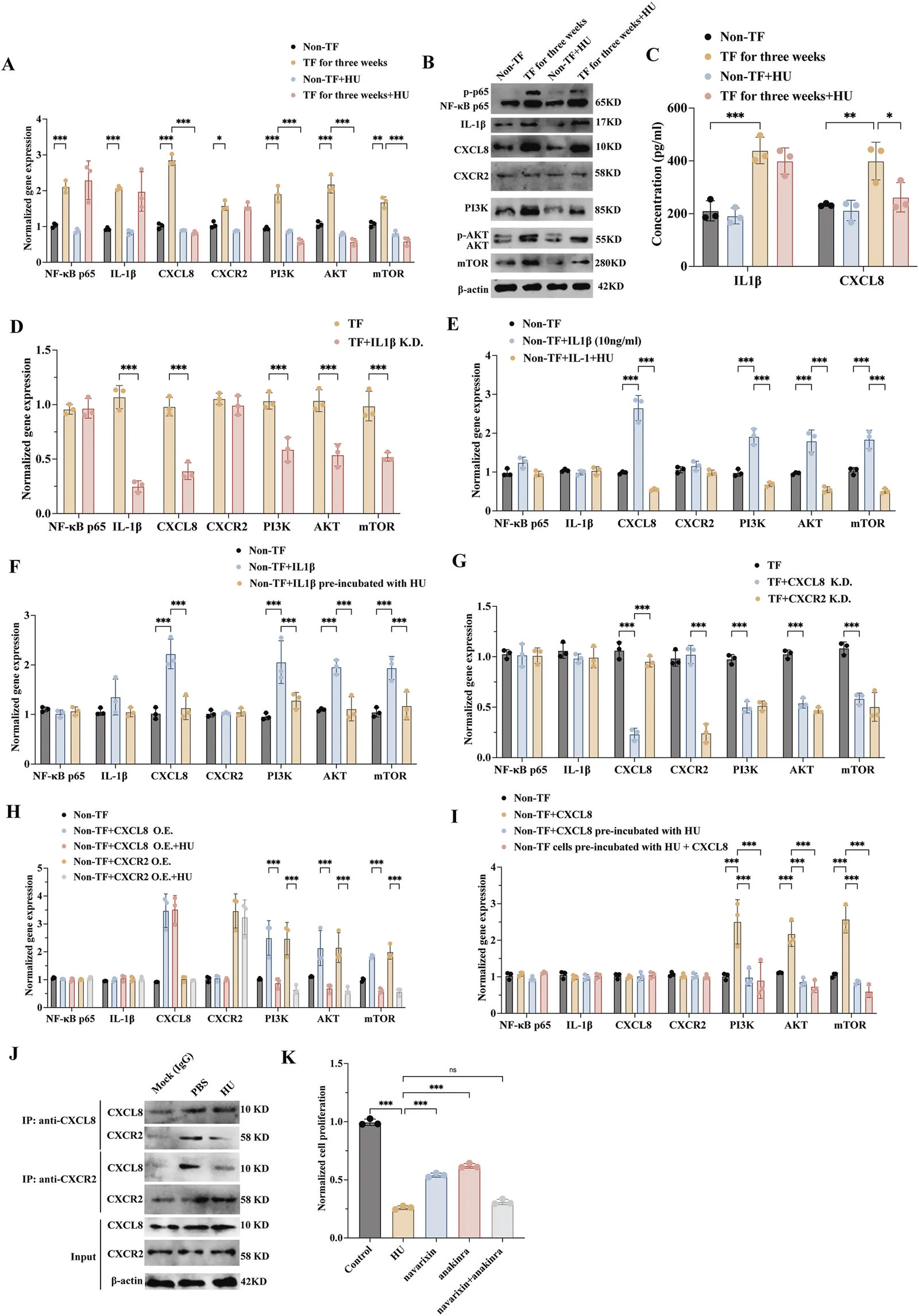
Effects of HU on axis-associated signaling and CXCL8–CXCR2 binding. **(A–C)** TF increases axis-associated readouts; HU reduces CXCL8 mRNA expression and secretion without affecting NF-κB p65, IL1β, or CXCR2 protein levels. **(D)** IL1Β knockdown reduces CXCL8 expression and downstream pathway readouts. **(E,F)** HU reduces responses to exogenous IL1β **(E)** and IL1β-induced responses after pre-incubation **(F)**. **(G)** CXCL8 or CXCR2 knockdown reduces the measured downstream pathway readouts. **(H,I)** HU reduces pathway readouts induced by CXCL8/CXCR2 overexpression **(H)** or exogenous CXCL8 **(I)**. **(J)** Co-IP shows reduced detection of the CXCL8-CXCR2 interaction. **(K)** Comparison of HU (30 μM) with navarixin (30 μM) and anakinra (30 μM), alone or in combination, in HCT116 viability assays under TF conditions at 48 h. Treatments were compared at the same nominal concentration and exposure duration. *P < 0.05, **P < 0.01, ***P < 0.001.

To test whether HU’s multi-target engagement confers a functional advantage over single-node inhibition, we compared HU against the CXCR2 antagonist navarixin and the IL-1 receptor antagonist anakinra in HCT116 viability assays under TF conditions. Treatments were compared at the same nominal concentration (30 μM) and exposure duration (48 h) to ensure a fair comparison. HU reduced cell viability to 26% ± 1.5%, a greater reduction than with navarixin alone (54% ± 2.2%, P < 0.01) or anakinra alone (62% ± 1.9%, P < 0.01), with the navarixin plus anakinra combination yielding a viability of 31% ± 2.3% (P < 0.01) (Figure 8K). We note that these are single-concentration comparisons, and full dose-response curves for combination treatments would be informative in future studies. These results support further evaluation of HU against the comparator treatments, due to simultaneous engagement of multiple axis nodes causes greater cell viability inhibition to colorectal cancer cells.

### 3.7 HU decreases M2-associated and increases M1-associated macrophage markers

To examine macrophage marker responses in a diabetic-mimetic setting, we exposed THP-1-derived macrophages to conditioned medium from HCT116 cultures. Conditioned medium from TF-exposed HCT116 cells increased M2-associated markers in THP-1-derived macrophages (Figures 9A,B) while decreasing M1-associated markers (Figures 9C,D). HU treatment, whether applied to the culture of TF-exposed HCT116 cells (TF + HU) or added directly to the macrophage culture (TF-exposed + HU), significantly reduced M2-associated markers (Figures 9E,F). Notably, HU not only reduced M2-associated marker expression but also increased M1-associated markers (Figures 9G,H). These marker changes were also examined following cytokine stimulation: exogenous addition of IL1β and CXCL8 to THP-1 cultures also increased M2-associated markers (Supplementary Figure S7A,B), but TF treatment further increased these markers, again favoring an M2-like marker profile (Supplementary Figure S7A,B). Importantly, HU also counteracted the increase in M2-associated macrophage marker expression induced by exogenous IL1β+CXCL8 and by TF exposure (Supplementary Figure S7A,B). Collectively, these findings show that HU reduces tumor cell-associated phenotypes and shifts macrophage marker expression in this diabetic-mimetic model.

**FIGURE 9:**
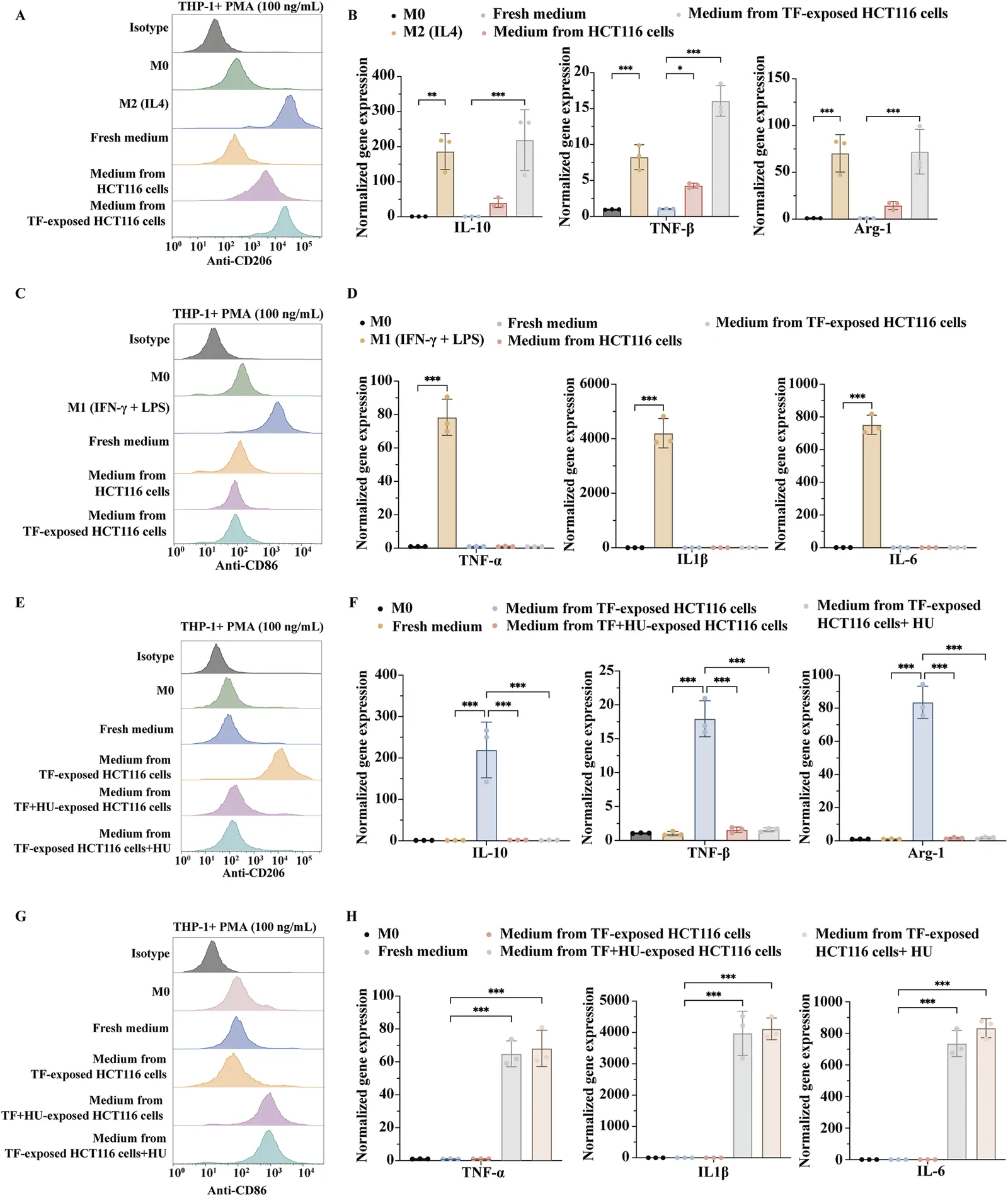
HU decreases M2-associated and increases M1-associated macrophage markers under diabetic-mimetic conditions. **(A,B)** TF-conditioned medium increases M2-associated markers relative to control, shown by CD206 flow cytometry **(A)** and M2 marker qRT-PCR (IL10, TGFB, ARG1) **(B)**. **(C,D)** TF decreases M1-associated markers relative to control, shown by CD86 flow cytometry **(C)** and M1 marker qRT-PCR (TNF, IL1Β, IL6) **(D)**. **(E,F)** HU treatment counteracts TF-induced M2-associated marker expression, shown by CD206 **(E)** and M2 markers **(F)**. **(G,H)** HU treatment increases M1-associated marker expression, shown by CD86 **(G)** and M1 markers **(H)**. *P < 0.05, **P < 0.01, ***P < 0.001.

## 4 Discussion

Diabetes and colorectal cancer intersect at a molecular interface that current therapies do not specifically address, an important clinical challenge given the substantial burden of cancer mortality among people with diabetes (Pearson-Stuttard et al., 2021). In this study, we combined bioinformatic discovery, deep learning- and structure-based virtual screening, and cellular assays to identify the IL1β-CXCL8-CXCR2 inflammatory-chemokine axis as a candidate module linking diabetes-related molecular features to the CRC immune microenvironment, and to characterize Hupehenine (HU), a steroidal alkaloid from Fritillaria hupehensis, as a natural multi-target ligand of this axis. Our results show that HU binds all three proteins (IL1β, CXCL8, and CXCR2) *in vitro* and shifts macrophage markers from an M2-like to an M1-like profile in a diabetic-mimetic conditioned-medium system. These findings provide *in vitro* evidence linking diabetic metabolic stress to immune-modulatory signaling in CRC, and suggest that polypharmacological targeting of the IL1β-CXCL8-CXCR2 axis warrants further investigation.

Expression of genes in the IL1β-CXCL8-CXCR2 axis was associated with immune infiltration and inhibitory immune-related transcriptional signatures. This pattern may be compatible with an immune-infiltrated microenvironment that also expresses inhibitory markers, as discussed in established TME classification frameworks (Chen and Mellman, 2017). In the single-cell analysis, the proportion of CXCL8-expressing myeloid cells correlated with both the M2-associated score (r = 0.667, P < 0.001) and the M1-associated score (r = 0.557, P < 0.001) (Figures 3M,N). The difference between these correlations was not formally tested; their relative magnitudes therefore do not demonstrate preferential M2 polarization or explain the survival association. No statistically significant difference in the measured CD8^+^ T-cell abundance was detected between the expression-defined groups (Figures 3O,P), which neither establishes nor excludes an effect on T-cell recruitment. Several hypotheses may explain the association between higher axis expression and longer overall survival in TCGA. MMR deficiency-associated immune features, including neoantigen-related responses, could contribute to a favorable immune context. Pre-existing CD8^+^ T cells or other immune populations, such as NK and γδ T cells, could also contribute, while higher IL1Β and CXCL8 expression may reflect an ongoing inflammatory response rather than a direct cause of improved survival. These explanations remain tested in the further study. MMRd tumors had higher CD8^+^ T-cell exhaustion-associated transcriptional scores (P = 0.0071) and M2-associated macrophage scores (P = 0.0138) than MMRp tumors (Figures 3Q,R); however, these scores measure gene-expression patterns, not T-cell dysfunction or macrophage suppressive activity. The reported involvement of CXCL8-CXCR2 signaling in myeloid recruitment and polarization provides a biological rationale for further investigation (Cambier et al., 2023; Korbecki et al., 2022). In our experiments, HU decreased M2-associated and increased M1-associated surface and transcript markers, demonstrating that the measured macrophage marker profile can be pharmacologically modulated under diabetic-mimetic conditions. Although these changes do not establish functional repolarization, they complement the transcriptomic findings and support the IL1β-CXCL8-CXCR2 axis as a candidate link between metabolic stress and the CRC immune microenvironment. Elucidating the relationships among axis expression, MMR status, immune-cell function, and survival requires multivariable adjustment, independent clinical validation, and direct functional studies.

A central rationale for pursuing HU was the hypothesis that simultaneously engaging multiple nodes of the IL1β-CXCL8-CXCR2 axis could produce effects distinct from inhibition of a single target. Our head-to-head comparison provides preliminary observations relevant to this hypothesis: HU suppressed HCT116 cell viability more effectively than the CXCR2 antagonist navarixin alone or the IL-1 receptor antagonist anakinra alone under TF conditions, with viability values numerically close to those observed for the navarixin plus anakinra combination (Figure 8K). This finding is consistent with a contribution from HU’s multi-target binding profile, which encompasses CXCL8, CXCR2, and the upstream mediator IL1β. Differences in potency, target occupancy, or off-target activity could also contribute. These data, while obtained in a single cell line and at a single time point, provide a rationale for further testing of the multi-target concept that complements the theoretical arguments outlined below.

The mechanistic rationale for multi-target intervention in this axis rests on two well-characterized properties of inflammatory signaling networks (Nogales et al., 2022; Stefan and Rafehi, 2024). First, cytokine networks exhibit extensive redundancy: IL-1 inhibition with anakinra or canakinumab may be limited by disease context and compensatory inflammatory pathways (Wang et al., 2025), and TNF-α inhibitors can provoke paradoxical immunological switching (De Stefano et al., 2023). In the present axis, CXCR2 blockade would not necessarily suppress upstream NF-κB/IL1β signaling or CXCL8 production (Figures 8A–C). HU’s ability to bind IL1β and reduce IL1β-induced responses in pre-incubation assays (Figure 8F) suggests it may influence responses to this upstream mediator, although we acknowledge that the affinity for IL1β (KD ∼12.7 μM) is substantially weaker than for CXCR2 (KD ∼505 nM), and the functional significance of this interaction requires further study. Second, chemokine systems exhibit ligand-receptor promiscuity (Eiger et al., 2021): CXCR2 is activated by at least seven ligands (CXCL1-3, CXCL5-8), and CXCL8 signals through both CXCR2 and CXCR1 (Cambier et al., 2023). A CXCR2-selective antagonist therefore leaves CXCR1-mediated CXCL8 signaling intact, and a pure anti-CXCL8 strategy does not address other CXCR2 ligands. HU’s dual ligand-plus-receptor binding profile, binding CXCL8 in SPR assays (Figure 6B) while interfering with CXCL8–CXCR2 binding (Figures 6F,G; Figure 8J), could potentially influence more than one signaling route. Whether HU inhibits CXCR1-mediated responses or signaling induced by other CXCR2 ligands remains to be determined.

The observed binding profile of HU motivates further investigation of its target spectrum. Network pharmacology analyses have shown that Fritillaria-derived steroidal alkaloids engage multiple inflammatory nodes (Kumar et al., 2025), and sipeimine, a closely related Fritillaria alkaloid, independently suppresses NLRP3 inflammasome-mediated IL1β production (Fang et al., 2024). Our overexpression experiments provide evidence consistent with an effect upstream of PI3K/AKT: NF-κB p65-driven proliferation and migration were attenuated by HU, whereas responses following PI3K/AKT overexpression were less sensitive to HU (Supplementary Figure S6A–G). These findings support involvement of the axis but do not define the complete target spectrum or exclude parallel mechanisms. Important caveats remain. The contribution of the weaker IL1β interaction (KD ∼12.7 μM) relative to the stronger CXCR2/CXCL8 blockade is unresolved; the reduction in IL1β-induced responses observed after pre-incubation (Figure 8F) suggests potential functional relevance, although the relationship between the concentrations used *in vitro* and achievable *in vivo* exposure is unknown. Furthermore, HU has not been profiled against nuclear hormone receptors, hERG channels, or a broader target panel, assessments that are necessary to determine whether the multi-target profile described here represents selective polypharmacology or broader promiscuity.

HU showed context-dependent activity *in vitro*: HCT116 cells chronically exposed to a diabetic-mimetic microenvironment exhibited approximately 2.7-fold greater sensitivity to HU than cells under standard conditions, whereas sensitivity to 5-fluorouracil and oxaliplatin was not enhanced. This difference may be related to TF-associated changes in the axis or other cellular responses, but its mechanism remains unresolved. Recent studies have examined links between diabetes-related metabolic conditions and tumor immunity: Peng et al. (2025) demonstrated that hyperglycemia drives CCL3-CCR1-mediated monocyte recruitment and subsequent M2 differentiation in colorectal liver metastases, while Wankhede et al. (2025) reported that the impact of type 2 diabetes on CRC survival varies according to tumor immune status at the population level (Peng et al., 2025; Wankhede et al., 2025). These studies support a broader link between metabolic stress and tumor immunity. We emphasize that HU, in its current form, is not a clinical candidate; its micromolar potency and uncharacterized pharmacokinetics place it at the lead-discovery stage. Structural optimization, informed by the growing number of Fritillaria alkaloid derivatives with defined structure-activity relationships (Kumar et al., 2025), or formulation-based delivery strategies may be needed to improve exposure, but pharmacokinetic studies are required to assess this possibility. The absence of a significant reduction in HCEC-1CT viability at concentrations up to 200 μM, together with the increased sensitivity of TF-conditioned CRC cells, supports further investigation of a potential *in vitro* selectivity window. These observations do not establish systemic safety or diabetes-specific efficacy.

Several limitations define the scope of this study. The diabetic-mimetic model captures key metabolic stressors but cannot replicate the complexity of an intact diabetic host. Macrophage characterization relied on a focused panel of surface and transcript markers rather than direct functional measurements. The observed marker changes do not establish functional repolarization. Direct assays of nitric oxide production, phagocytosis, and T-cell suppressive activity are needed. Similarly, exhaustion-associated transcriptional scores do not establish T-cell dysfunction, and computationally inferred cell communication does not demonstrate functional signaling. The causal contributions of individual HU targets to the marker changes also remain unresolved. The exploratory diabetes-related expression signature requires validation against clinical diabetes status. The survival associations also require multivariable adjustment and independent validation before prognostic or mechanistic conclusions can be drawn. Finally, the pharmacokinetic properties, oral bioavailability, and full target spectrum of HU remain to be characterized. Addressing these gaps, through immunocompetent diabetic CRC mouse models, comprehensive ADMET profiling, and medicinal chemistry exploration, will determine whether the multi-target hypothesis explored here can advance toward translational application.

## 5 Conclusion

In summary, this study prioritizes the IL1β-CXCL8-CXCR2 axis as a candidate immunomodulatory module at the diabetes-CRC interface. Hupehenine, a steroidal alkaloid from Fritillaria hupehensis, binds all three proteins *in vitro*, with the strongest measured affinity for CXCR2, and interferes with CXCL8-CXCR2 binding. In a diabetic-mimetic model, HU reduces tumor cell viability and motility and modulates macrophage markers. Single-concentration comparisons support further investigation of its pharmacological profile but do not establish the causal contribution of individual targets. Further studies should address macrophage function, *in vivo* efficacy, target selectivity, and pharmacokinetics.

## Data Availability

The datasets presented in this study can be found in online repositories. Repository details are provided at: https://figshare.com/s/20956a8398c1a821a428. Publicly available datasets analyzed include TCGA-COAD and TCGA-READ. Additional data are available from the corresponding authors upon reasonable request.

## Glossary

5-FU: 5-Fluorouracil
ADMET: Absorption, Distribution, Metabolism, Excretion, and Toxicity
AGE: Advanced Glycation End Product
AKT: Protein Kinase B (also known as PKB)
AP-1: Activator Protein 1
CCL3: C-C Motif Chemokine Ligand 3
CCR1: C-C Motif Chemokine Receptor 1
CD86: Cluster of Differentiation 86
CD206: Mannose Receptor (also known as MRC1)
Co-IP: Co-Immunoprecipitation
CRC: Colorectal Cancer
CXCL8: C-X-C Motif Chemokine Ligand 8 (Interleukin-8)
CXCR1: C-X-C Motif Chemokine Receptor 1
CXCR2: C-X-C Motif Chemokine Receptor 2
EMT: Epithelial-Mesenchymal Transition
EPC: Edge Percolation Component
FBS: Fetal Bovine Serum
GO: Gene Ontology
HCEC-1CT: Human Colonic Epithelial Cells
hERG: Human Ether-à-go-go-Related Gene
HU: Hupehenine
IC_50_: Half-Maximal Inhibitory Concentration
IL1β: Interleukin-1 Beta
IL6: Interleukin 6
IL10: Interleukin 10
KEGG: Kyoto Encyclopedia of Genes and Genomes
KD: Equilibrium Dissociation Constant
MAPK: Mitogen-Activated Protein Kinase
MCC: Maximum Clique Centrality
MCODE: Molecular Complex Detection
MDS: Molecular Dynamics Simulation
MDSC: Myeloid-Derived Suppressor Cell
MM/GBSA: Molecular Mechanics/Generalized Born Surface Area
MMR: Mismatch Repair
MMRd: Mismatch Repair Deficient
MMRp: Mismatch Repair Proficient
MNC: Maximum Neighborhood Component
NF-κB: Nuclear Factor Kappa B
NLRP3: NOD-Like Receptor Family Pyrin Domain Containing 3
OMIM: Online Mendelian Inheritance in Man
PCA: Principal Component Analysis
PI3K: Phosphoinositide 3-Kinase
PMA: Phorbol 12-Myristate 13-Acetate
PPI: Protein-Protein Interaction
qRT-PCR: Quantitative Real-Time Polymerase Chain Reaction
RAGE: Receptor for Advanced Glycation End Products
Rg: Radius of Gyration
RMSD: Root-Mean-Square Deviation
RMSF: Root-Mean-Square Fluctuation
SPR: Surface Plasmon Resonance
STRING: Search Tool for the Retrieval of Interacting Genes/Proteins
TCGA: The Cancer Genome Atlas
TF: Three Factors (High Glucose, Insulin, Palmitic Acid)
TGFB: Transforming Growth Factor Beta
TME: Tumor Microenvironment
TNF: Tumor Necrosis Factor
TNF-α: Tumor Necrosis Factor Alpha
TTD: Therapeutic Target Database
