# Supplementary material for: Hupehenine suppresses tumor cell phenotypes and modulates macrophage marker profiles in a diabeticmimetic colorectal cancer model
Source: Front Pharmacol. 2026 Oct 20;17:1962620. doi: 10.3389/fphar.2026.1962620 (PMC13727481; doi:10.3389/fphar.2026.1962620)
Supplement: Supplementary file 1 [file Supplementaryfile1.docx]

**Hupehenine, a Multi-Target Ligand of the IL1β–CXCL8–CXCR2 Axis, Suppresses Tumor Cell Phenotypes and Modulates Macrophage Marker Profiles in a Diabetic-Mimetic Colorectal Cancer Model**

Ming Qi Zhu^1,†^, Zi Tong Shi^2,†^, Hong Mei Hu^2^, Qi Dong Xue^2^, Xin Yu Gu^2^, Xin Cheng Li^2^, Qiu Yan Wang^2^, Yan Zhao^2^, You Wei Zhang^1^, Ji Zhong Zhao^2^

1: Xuzhou Clinical College of Xuzhou Medical University, Xuzhou Central Hospital, Xuzhou 221000, China

2: Lab of Cell Biology, School of Life Sciences, Jiangsu Normal University, Xuzhou, Jiangsu 221116, China

Correspondence: Drs. J.Z. Zhao and Y.W. Zhang, School of Life Sciences, Jiangsu Normal University, Xuzhou, Jiangsu 221116, PR China. Email: [jz_zhao@jsnu.edu.cn](mailto:jz_zhao@jsnu.edu.cn); Tel: 86-18260722902.

† These authors contributed equally to this work.

**Running title:** Hupehenine Targets IL1β-CXCL8-CXCR2 Axis in Diabetic CRC

**Supplementary Figures**

**Figure S1. Expression, prognostic value, and co-regulation of candidate hub genes.** (A–F) The mRNA levels of TNF, IL6, IFNG, TLR4, NFKB1, and IL10 show no significant differential expression between CRC tumors (n=275) and normal mucosa (n=349; TCGA-COAD/READ, log2(TPM+1)). TNF: P = 0.45; IL6: P = 0.34; IFNG: P = 0.09; TLR4: P = 0.12; NFκB1: P = 0.52; IL10: P = 0.06. (G–L) Kaplan-Meier survival analysis indicates that high expression of these six genes is not associated with overall survival in CRC patients. Sample size variations across panels are due to independent gene-level stratification and minor missing expression data in the TCGA dataset. (M, N) IL1B and CXCL8 show significant positive transcriptional correlations with CXCR2 (Pearson R = 0.61, P = 2.2 × 10⁻¹⁶, and R = 0.59, P = 2.2 × 10⁻¹⁶, respectively). (O) Bubble plot showing the expression of canonical marker genes across major cell clusters. The size of the dots represents the fraction of cells expressing the corresponding gene within each cluster, and the color intensity indicated the mean expression level.

**Figure S2. Expression distribution of the IL1β-CXCL8-CXCR2 axis genes across major cell clusters and Cell-cell communication analysis of the IL1β-CXCL8-CXCR2 axis.** (A) UMAP plot showing the top-level classification of major cell types, including epithelial cells (Epi), T/NK/innate lymphoid cells (TNKILC), myeloid cells, plasma cells, B cells, stromal cells (Strom), and mast cells. (B-D) UMAP feature plots showing the expression levels of IL1B (B), CXCL8 (C), and CXCR2 (D) across the cell clusters. (E) Heatmap showing the inferred intercellular communication scores (log(1+score)) based on CellChat (v1.6.0) analysis. The IL1β-CXCL8-CXCR2 signaling axis is primarily characterized by myeloid-to-myeloid communication (e.g., granulocytes and monocytes acting as major senders), consistent with the single-cell expression patterns.

**Figure S3 Rg and MM/GBSA.** (A) Binding energy profiles of HU with target proteins during the MDS calculations. (B-D) Radius of gyration.

**Figure S4. HU exhibits enhanced efficacy against SW480 colorectal cancer cells under TF conditions.** (A) TF treatment time-dependently increased SW480 cell proliferation. (B) HU showed greater inhibitory effects on cell viability under TF conditions compared to non-TF conditions (IC₅₀ = 26.7 ± 1.3 μM under TF vs. 72.2 ± 3.6 μM under non-TF conditions; MTT assay at 48 h). (C) The TF microenvironment potently activated the IL1β-CXCL8-CXCR2 signaling axis, as evidenced by upregulation of NF-κB p65, IL1β, CXCL8, CXCR2, PI3K, AKT, and mTOR. HU attenuated TF-induced upregulation of CXCL8, PI3K, AKT, and mTOR, while sparing NF-κB p65, IL1β, and CXCR2. *P < 0.05, **P < 0.01, ***P < 0.001.

**Figure S5.** **Genetic manipulation of key axis components in HCT116 cells.** (A-C) Successful knockdown of IL1B, CXCL8, and CXCR2 mRNA levels was achieved using specific siRNAs. (D-H) Overexpression of CXCL8, CXCR2, NFKB1 (p65), PIK3CA (PI3K), and AKT1 was confirmed in transfected HCT116 cells.

**Figure S6. HU inhibits tumor progression by targeting the IL1β-CXCL8-CXCR2 axis.** (A) NF-κB p65 overexpression activates the axis and downstream effectors (overexpression validation shown in Fig. S5F). (B–D) HU counters NF-κB p65-driven proliferation (B) and migration (C, D). (E–G) HU shows minimal effect on malignancy driven by downstream PI3K/AKT overexpression (overexpression validation shown in Fig. S5G and S5H). *P < 0.05, **P < 0.01, ***P < 0.001.

Figure S7. HU counteracts exogenous cytokine-induced M2 surface marker expression in a diabetic context. (A and B) Flow cytometric analysis (A) and RT-qPCR (B) demonstrate that the combination of IL-1β and CXCL8 promotes macrophage M2 polarization (increased CD206, IL10, TGFB, and ARG1), an effect which is enhanced by concurrent diabetic mimetic (TF) exposure. Treatment with HU effectively counteracts this TF-amplified, cytokine-driven M2 marker expression.

**Supplementary Methods**

**Identification of Genes Co-expressed in Diabetes and Colorectal Cancer**

To systematically delineate the shared molecular landscape between diabetes and CRC, an integrated bioinformatics screening strategy was employed. Disease-associated genes were comprehensively retrieved from four authoritative public databases: GeneCards, DisGeNET, the Online Mendelian Inheritance in Man (OMIM), and the Therapeutic Target Database (TTD). Systematic queries were performed using relevant keywords including "diabetes mellitus", "type 2 diabetes", "colorectal cancer", and "colon cancer" to ensure broad coverage. For the GeneCards database, a relevance score threshold of ≥1 was applied to filter results. For the DisGeNET database, gene-disease association scores were filtered with thresholds of ≥0.02 for diabetes and ≥0.01 for CRC to ensure biological relevance. Genes uniquely associated with either DM or CRC were independently compiled from each source. The compiled lists from all four databases were then merged, and duplicate entries were removed, resulting in a non-redundant master list of 1043 DM-associated genes and 3632 CRC-associated genes (**Table S1**). Finally, to identify genes concurrently implicated in both pathologies, the intersection of the diabetes gene set and the CRC gene set was calculated, yielding 531 candidate shared genes for subsequent analysis (**Table S2**). URLs for all accessed resources are provided in **Table S3**.

**Functional and Pathway Enrichment Analysis**

To elucidate the functional significance and pathway involvement of the 531 co-expressed genes identified at the diabetes-CRC interface, we performed systematic Gene Ontology (GO) and Kyoto Encyclopedia of Genes and Genomes (KEGG) enrichment analyses. The official gene symbols were first converted to standardized Entrez IDs. Enrichment was then executed using the clusterProfiler package (version 4.3.2) in R, with a statistical significance cutoff set at an adjusted p-value < 0.05. Significant GO terms, categorized into Biological Processes (BP), Cellular Components (CC), and Molecular Functions (MF), were summarized and visualized. The distribution of the most significantly enriched terms across these three ontologies was presented in a bar plot. Concurrently, the key KEGG pathways enriched among the gene set were illustrated in a scatter plot, depicting the gene ratio and statistical significance for each pathway. All web resources and tools utilized in this analytical workflow are documented in **Table S3**.

**Construction of an Integrated Interaction Network**

To synthesize the complex relationships between the shared genes and their enriched functional terms, a comprehensive systems biology network was constructed using Cytoscape software (version 3.6.0). The network integrated two core components: 1) the 531 shared diabetes-CRC genes, and 2) the top 20 most significantly enriched terms from each of the three GO categories (BP, CC, MF) and the KEGG pathway analysis. This integrative approach provided a visual and topological framework representing the functional landscape linking diabetic pathology to colorectal carcinogenesis.

**Identification of Hub Genes Bridging Diabetes and Colorectal Cancer**

A multi-step bioinformatic workflow was employed to identify central molecular regulators (hub genes) from the shared diabetes-CRC gene set. First, a protein-protein interaction (PPI) network was constructed using the 531 shared genes as input in the STRING database (v11.5, confidence threshold ≥ 0.4) and visualized in Cytoscape (v3.6.0). To identify densely connected functional modules, the network was analyzed using the Molecular Complex Detection (MCODE) algorithm (default parameters), yielding a preliminary candidate list of 16 intramodular hub genes. Subsequently, the most topologically influential nodes across the entire network were robustly defined using the CytoHubba plugin, applying four distinct centrality algorithms—Degree (measuring direct connections), Edge Percolation Component (EPC) (robustness-based), Maximum Clique Centrality (MCC) (density-weighted clustering), and Maximum Neighborhood Component (MNC) (local network size) —to independently rank genes. The top 20 genes from each algorithm were integrated, and a consensus analysis of these rankings was performed. The intersection of results from all four methods, visualized via an UpSet plot, was used to define the highest-confidence core hub genes.

**Diabetes Risk Stratification**

Diabetes risk stratification was performed using a composite 14-gene signature developed in this study. First, the 531 genes co-associated with diabetes and CRC were subjected to LASSO (Least Absolute Shrinkage and Selection Operator) Cox regression analysis to identify the most prognostically relevant genes. The LASSO penalty parameter λ was selected via 10-fold cross-validation using the minimum partial likelihood deviance criterion. This analysis yielded a 14-gene signature (TCF7L2, PPARG, ADIPOQ, LEP, IGF1, IGF2, IRS1, IRS2, SLC30A8, CDKAL1, GCK, PDK4, IL6, TNF), with each gene assigned a non-zero regression coefficient. A risk score was then calculated for each patient using the following formula:

$$\text{Risk Score}=\sum_{i=1}^{14} \left( \text{Expression}_{i}\times\text{Coefficient}_{i} \right)$$

where Expression*_i_* represents the normalized expression level of the *i*-th gene, and Coefficient*_i_* represents the LASSO-derived regression coefficient for that gene. Patients from the TCGA-COAD and READ cohorts were subsequently dichotomized into high-diabetes risk (high-DM) and low-diabetes risk (low-DM) groups using the median risk score as the cutoff value. The performance of this 14-gene signature was evaluated using Kaplan-Meier survival analysis and receiver operating characteristic (ROC) curve analysis in the training cohort.

**AI-Driven Virtual Screening for Multi-Target Compound Identification**

To discover natural compounds capable of simultaneously engaging the IL1β, CXCL8, and CXCR2 targets, a deep learning-based virtual screening was conducted using the DeepPurpose toolkit (https://github.com/kexinhuang12345/DeepPurpose), a specialized library for drug-target interaction (DTI) prediction and virtual screening. We leveraged its framework to construct a multi-target prediction model tailored to our screening objective. The molecular structures of all compounds were obtained from MCE natural product library and encoded using the 'Morgan' fingerprint representation, a widely adopted cheminformatics descriptor. A customized DTI prediction model was then configured within DeepPurpose to predict the interaction affinities of each compound against each of the three protein targets (IL1β, CXCL8, and CXCR2) concurrently. The screening process generated a unified ranked list of compounds based on a composite score reflecting their predicted binding potential across all three targets. From this list, the top 30 candidate compounds exhibiting the highest multi-target affinity scores were selected for subsequent consensus analysis with structure-based screening results. In parallel, a structure-based virtual screening was conducted using AutoDock Vina the Autodock vina‌. The natural product library was docked into the prepared binding sites of the three target proteins, and the workflow generated a ranked list of its top 30 hits based on docking scores. The results from these two independent screening methods were cross-referenced to identify a consensus set of compounds. A compound was considered a consensus hit if it appeared in both the DeepPurpose top-30 list and the AutoDock Vina Autodock vina‌ top-30 list. This analysis yielded a final compound, HU (HY-N0413), which exhibited high potential for binding with all three targets.

**Verification of the binding model using MDS**

Trajectories were post-processed with gmx trjconv. Molecules were made whole and packed into the unit cell using the protein as the centring group (-center -pbc mol -ur compact); a subsequent jump-removal pass (-pbc nojump) was applied to eliminate periodic-image discontinuities; frames were finally superposed on the protein backbone (-fit rot+trans). All RMSD values were computed with a single common least-squares fit group (backbone), varying only the group used for RMSD evaluation, so that protein, ligand and complex curves are directly comparable; the reference structure was the first frame of the production run. Root-mean-square fluctuations were computed per residue for the protein backbone with gmx rmsf -res. Ligand retention was quantified as the minimum ligand–protein distance using gmx mindist, evaluated both with the default minimum-image convention and with -nopbc on the jump-corrected trajectory, which distinguishes genuine unbinding from periodic-image artefacts. The radius of gyration was calculated with gmx gyrate. Principal component analysis of backbone fluctuations was performed with gmx covar and gmx anaeig using the first two eigenvectors, and two-dimensional and three-dimensional Gibbs free-energy landscapes were obtained from the PC1–PC2 projection with gmx sham. Binding free energies were calculated with the MM/GBSA method implemented in gmx_MMPBSA 1.6.4 (based on MMPBSA.py v16.0) using 101 evenly spaced frames extracted from each trajectory, with the receptor and ligand defined by the residue masks :1–150 (IL-1β), :1–135 (CXCL8) and :1–298 (CXCR2) and residue 151/136/299 as the ligand, respectively. All software resources and core GROMACS commands are documented in **Tables S3** and **S4**, respectively.

**Verification of HU-target binding via molecular docking**

To validate the predicted interactions, the binding of HU to IL1β, CXCL8, and CXCR2 was assessed through molecular docking simulations. The three-dimensional structure of HU (PubChem CID: 14240934) was acquired in SDF format and subsequently converted to PDB format. The crystal structures of the target proteins were obtained from the Protein Data Bank (PDB IDs: 1HIB for IL1β, 1ICW for CXCL8, and 8XWN for CXCR2). All protein structures were prepared for docking by removing non-essential water molecules and co-crystallized ligands using PyMOL (v2.3.4). Essential hydrogen atoms and Gasteiger partial charges were then added using AutoDock Tools (v1.5.7). Finally, both the prepared ligand and receptor files were converted into the required PDBQT format. Docking calculations were performed using AutoDock4 (v4.2.6), and the resulting binding conformations were analyzed and visualized with Discovery Studio 2019.

**Surface Plasmon Resonance (SPR) Binding Analysis**

Direct and competitive binding interactions were characterized using real-time, label-free SPR spectroscopy on a Biacore™ 1K instrument. Recombinant human CXCR2 (extracellular domain) was immobilized on a Series S CM5 sensor chip via standard amine-coupling chemistry, using EDC/NHS activation to achieve a density of ~8000 response units (RU), with ethanolamine blocking. For direct binding kinetics, serially diluted HU (in 1×PBS-P+running buffer with 5% DMSO) was injected over the CXCR2 surface and reference cell at 10 µL/min (0 s association/62 s dissociation) in a multi-cycle format. In reverse configurations, IL1β or CXCL8 were separately immobilized, and HU served as the analyte. The binding of natural ligand-receptor pairs (IL1β or CXCL8 to immobilized CXCR2) was similarly assessed. All sensorgrams were double-referenced and globally fitted to a 1:1 Langmuir binding model to derive kinetic rate constants (K_a_, K_d_) and the equilibrium dissociation constant (K_D_). For competitive inhibition, the CXCR2 surface was first pre-saturated with 10 µM HU, followed by immediate co-injection of IL1β or CXCL8 without surface regeneration, and the resulting binding response was compared to the uninhibited baseline.

**Chemicals, Reagents, cells and antibodies**

Chemically defined compounds HU (Cat# HY-N0413) were purchased from MedChemExpress, Shanghai, China. Its purity reached over 99 %, which were dissolved in dimethyl sulfoxide (DMSO) and kept at 4 ^o^C till use. Methyl thiazolyl tetrazolium (MTT) were purchased from Sigma-Aldrich (M2128; St. Louis, MO, USA). Antibodies included: anti-NF-κB p65 (Cat# 80979-1-RR, rabbit), anti-IL1β (Cat# 16806-1-AP, rabbit), anti-PI3K (Cat# 20584-1-AP, rabbit), anti-AKT (Cat# 10176-2-AP, rabbit), anti-CXCL8 (Cat# 27095-1-AP, rabbit), anti-mTOR (Cat# 20657-1-AP, rabbit), and anti-β-actin (Cat# 66009-1-Ig, mouse) from Proteintech (Wuhan, Hubei, China); PE anti-human CD68 (Cat# 333807, mouse), APC anti-human CD206 (Cat# 333807, mouse) and Alexa Fluor 488 anti-human CD86 (Cat# 305413, mouse) from BioLegend (Beijing, China). Secondary antibodies were goat anti-rabbit (Cat# SA00001-2) and anti-mouse (Cat# SA00001-1) both from Proteintech. The human CRC cell lines HCT116 and SW480 and the acute monocytic leukemia cell line THP-1 were procured from the American Type Culture Collection (ATCC, Manassas, VA, USA). The non-tumorigenic human colonic epithelial cell line HCEC-1CT was acquired from YaJi Biological (Shanghai, China).

**Cell Culture and treatment**

The HCT116 and HCEC-1CT cells were maintained in Dulbecco’s Modified Eagle’s Medium (DMEM) supplemented with 10% (v/v) heat-inactivated fetal bovine serum (FBS) and 1% penicillin/streptomycin. Cells were grown at 37°C in a humidified atmosphere of 5% CO₂. For all experiments, cells were seeded and allowed to adhere until reaching approximately 70-80% confluence prior to treatment. Stock solutions of test compounds were prepared in dimethyl sulfoxide (DMSO) and diluted directly into the culture medium to achieve the final working concentrations; an equivalent volume of DMSO (≤0.1%) was added to vehicle control cultures. Following treatment, cells were harvested at specified time points: samples for mRNA analysis were collected at 36 hours, while those for cell viability assays and western blot analysis were processed at 48 hours post-treatment.

THP-1 monocytic cells were cultured in Roswell Park Memorial Institute (RPMI) 1640 medium supplemented with 10% fetal bovine serum (FBS). To differentiate monocytes into macrophages, cells were seeded at a density of 5.0 × 10⁵ cells per well in 12-well suspension culture plates and stimulated with 50 ng mL⁻¹ phorbol 12-myristate 13-acetate (PMA) for 48 hours. Following differentiation, the adherent macrophages were gently washed twice with phosphate-buffered saline (PBS) and subsequently incubated for 24 hours with conditioned media collected from the following groups: untreated HCT116 cells, HCT116 cells exposed to the TF diabetic mimetic, HCT116 cells exposed to both TF and HU (here termed “TF + HU (co-treatment)”; HU and TF applied together to the HCT116 culture), or a mixture of conditioned medium from TF-exposed HCT116 cells with HU added directly to the macrophage culture (here termed “TF-conditioned medium + HU (macrophage exposure)”). For polarization controls, macrophages were stimulated with 10 ng mL⁻¹ LPS plus 20 ng mL⁻¹ IFN-γ to induce M1 polarization, or with 20 ng mL⁻¹ IL-4 to induce M2 polarization. After the treatment period, cells were harvested for subsequent polarization analysis.

**Cell viability Assay**

Cells were seeded in 96-well plates at a density of 6,000 cells per well and allowed to adhere for 24 hours prior to treatment. Following a 48-hour incubation with the test compounds, 10 µL of MTT reagent (5 mg/mL in PBS) was added to each well. The plates were then incubated at 37°C for 4 hours to allow formazan crystal formation. Subsequently, the culture medium was carefully removed, and the insoluble formazan crystals were dissolved in 150 µL of dimethyl sulfoxide (DMSO) with gentle agitation for 10-15 minutes at room temperature. The absorbance of the solution in each well was measured at a wavelength of 490 nm using a microplate spectrophotometer. Cell viability was expressed as a percentage relative to the vehicle-treated control group.

**RNA extraction and quantitative real-time PCR (qRT-PCR) Analysis**

Total RNA was isolated from treated cells using TRIzol reagent, and its concentration and purity were assessed spectrophotometrically. For cDNA synthesis, 1 µg of total RNA was reverse-transcribed using M-MLV Reverse Transcriptase with oligo(dT) primers. Quantitative real-time PCR was performed on a‌ 7300Plus Real-Time PCR System (ThermoFisher, Waltham, MA, USA) using TransStart Top Green qPCR SuperMix (Cat# AQ131–01; Transgene Biotech, Beijing, China). The amplification protocol consisted of an initial denaturation at 95°C for 30 seconds, followed by 40 cycles of 95°C for 5 seconds and 60°C for 30 seconds. All reactions were run in triplicate. The relative mRNA expression levels of target genes were normalized to the housekeeping gene GAPDH and calculated using the 2^−ΔΔCt^ method. The sequences of all gene-specific primers used in this study are listed in **Table S5**.

**Western Blot Analysis**

For protein extraction, treated cells were washed with ice-cold PBS and lysed using RIPA buffer (25 mM Tris-HCl, pH 7.4, 150 mM NaCl, 1 mM EDTA, 1% NP-40, 5% glycerol) supplemented with a protease inhibitor cocktail. The lysates were clarified by centrifugation at 13,000 × g for 30 minutes at 4°C. The protein concentration in the supernatant was determined with a BCA protein assay kit. Equal amounts of protein were separated by SDS-polyacrylamide gel electrophoresis and subsequently transferred onto a polyvinylidene difluoride (PVDF) membrane. The membrane was blocked with 5% non-fat milk in TBST and then incubated overnight at 4°C with specific primary antibodies. After washing, the membrane was incubated with an appropriate horseradish peroxidase (HRP)-conjugated secondary antibody. Protein bands were visualized using an enhanced chemiluminescence (ECL) substrate and imaged with a chemiluminescence imaging system. Band intensity was quantified using ImageJ software.

**Overexpression of protein using plasmid**

The full-length coding sequences of target genes were amplified by PCR from human cDNA using gene-specific primers (**Table S6**) and subsequently cloned into the pcDNA3.1(+) expression vector via *Hind III* and *EcoR I* restriction sites. The constructed plasmids were transformed into *E. coli* DH5α competent cells for amplification, followed by extraction and purification using a plasmid miniprep kit. All plasmid sequences were verified by sequencing through Genomics institution (BGI, Beijing, China). For transient transfection, HCT116 cells were seeded and grown to 60-70% confluence. Plasmid DNA and Lipofectamine 2000 (Cat# 11668030; Thermo Fisher Scientific, MA, USA) transfection reagent was separately diluted in DMEM reduced serum medium, incubated for 5 minutes, combined, and incubated for an additional 20 minutes at room temperature to form complexes. The DNA-lipid complexes were then added dropwise to the cell culture medium. After 6 hours, the transfection mixture was replaced with fresh complete medium, and cells were cultured for an additional 36 hours before harvest. Transfection efficiency and overexpression of the target genes were confirmed at both mRNA and protein levels by qRT-PCR and western blot analysis, respectively.

**Gene knockdown using small interfering RNA (siRNA)**

For gene silencing, HCT116 cells were seeded in six-well plates at a density of 5 × 10⁴ cells per well and cultured for 24 hours. Cells were then transfected with 50 nM of gene-specific siRNA oligonucleotides (GenePharam, Shanghai, China) targeting IL1β, CXCL8, CXCR2, or a non-targeting control siRNA, using a commercial siRNA transfection reagent according to the manufacturer's protocol. Briefly, the siRNA and transfection reagent were separately diluted in serum-free medium, combined, and incubated to form complexes before being added to the cell culture. After 6 hours, the transfection mixture was replaced with fresh complete medium. Cells were harvested 24 hours post-transfection, and the efficiency of protein knockdown was evaluated by western blot analysis. The sequences of all siRNA oligonucleotides used are provided in **Table S7**.

**Flow cytometric analysis of macrophage polarization**

To quantify macrophage polarization, THP-1-derived macrophages were harvested after 24 hours of treatment by gentle scraping, washed with PBS for twice, and stained for surface and intracellular markers. Cells were first incubated with fluorescently conjugated antibodies against surface markers CD86 (M1-associated) and CD206 (M2-associated) for 30 minutes at 4°C in the dark. Appropriate isotype controls were included for each antibody. After a final wash, cells were resuspended in PBS and analyzed immediately on a flow cytometer (ACEA Bioscience, San Diego, CA, USA). Data were collected for a minimum of 10,000 events per sample. The resulting fluorescence data were analyzed using FlowJo software (v10.8.1), and the mean fluorescence intensity (MFI) for each marker was calculated to determine the M1/M2 polarization status.
